# Improving airport security with IoT-powered deep learning methods for threat detection and intelligent recommendation systems

**DOI:** 10.1038/s41598-026-54104-z

**Published:** 2026-05-28

**Authors:** Mohamed Basem, John Zaki, M. Sabry Saraya, Fatma M. Talaat

**Affiliations:** 1 Computer and Control Systems Engineering, Faculty of Engineering, Mansoura University, Mansoura, Egypt; 2 Software Engineering Department, Faculty of Informatics, German International University, Cairo, Egypt; 3 Faculty of Artificial Intelligence, Kafrelsheikh University, Kafrelsheikh, 33516 Egypt

**Keywords:** Airport security, IoT, Deep learning, Recommendation systems, Engineering, Mathematics and computing

## Abstract

This study introduces a novel framework that integrates deep learning algorithms, intelligent recommendation systems, and Internet-of-things (IoT) devices to improve airport security. Surveillance cameras are used by the system to identify potentially dangerous activities, such leaving luggage unattended. By analyzing interactions and movement patterns, anomalies can be found, and security staff can receive notifications. To improve security measures based on historical data and current information, it makes use of recommendation systems. This integrated approach reduces false alerts, improves operating efficiency, and strengthens security safeguards. Case studies and simulations confirm the framework’s effectiveness. Interestingly, transfer learning using MVCNN architecture detected threats in test data that had never been seen before with an accuracy of above 95%. Additionally, the suggested ISODI approach outperformed Decision Tree and K-Nearest Neighbors in identifying anomalies associated with aircraft delays, with an accuracy of 0.99. These results show how the framework can raise airport security requirements. Notwithstanding the beneficial developments of this paradigm, more investigation is necessary to identify the best use case for it in the security systems in place today and to handle any privacy issues that may arise. To handle the complexities of various airport environments, practical systems would require extensive testing and adaptability.

## Introduction

The aviation sector faces both opportunities and challenges because of the growing volume of air travel. Even if it stands for global connectedness and economic prosperity, security measures must also be continuously improved to keep up with the ever-changing dangers. It is difficult for traditional security systems, which usually rely on permanent equipment and human supervision, to efficiently monitor large airport terminals and quickly identify suspect activity. This demonstrates a serious weakness in airport security, which could endanger the security of travelers, staff, and property.

A revolutionary approach to security solutions that make use of new technology is necessary to meet this important challenge. New developments in deep learning algorithms and Internet-of-things (IoT) devices provide encouraging prospects for enhancing airport security. IoT devices transform physical spaces into networks of linked sensors, enabling real-time data collection and supervision of various aspects of an airport, such as baggage handling and passenger flow.

**Fig. 1:**
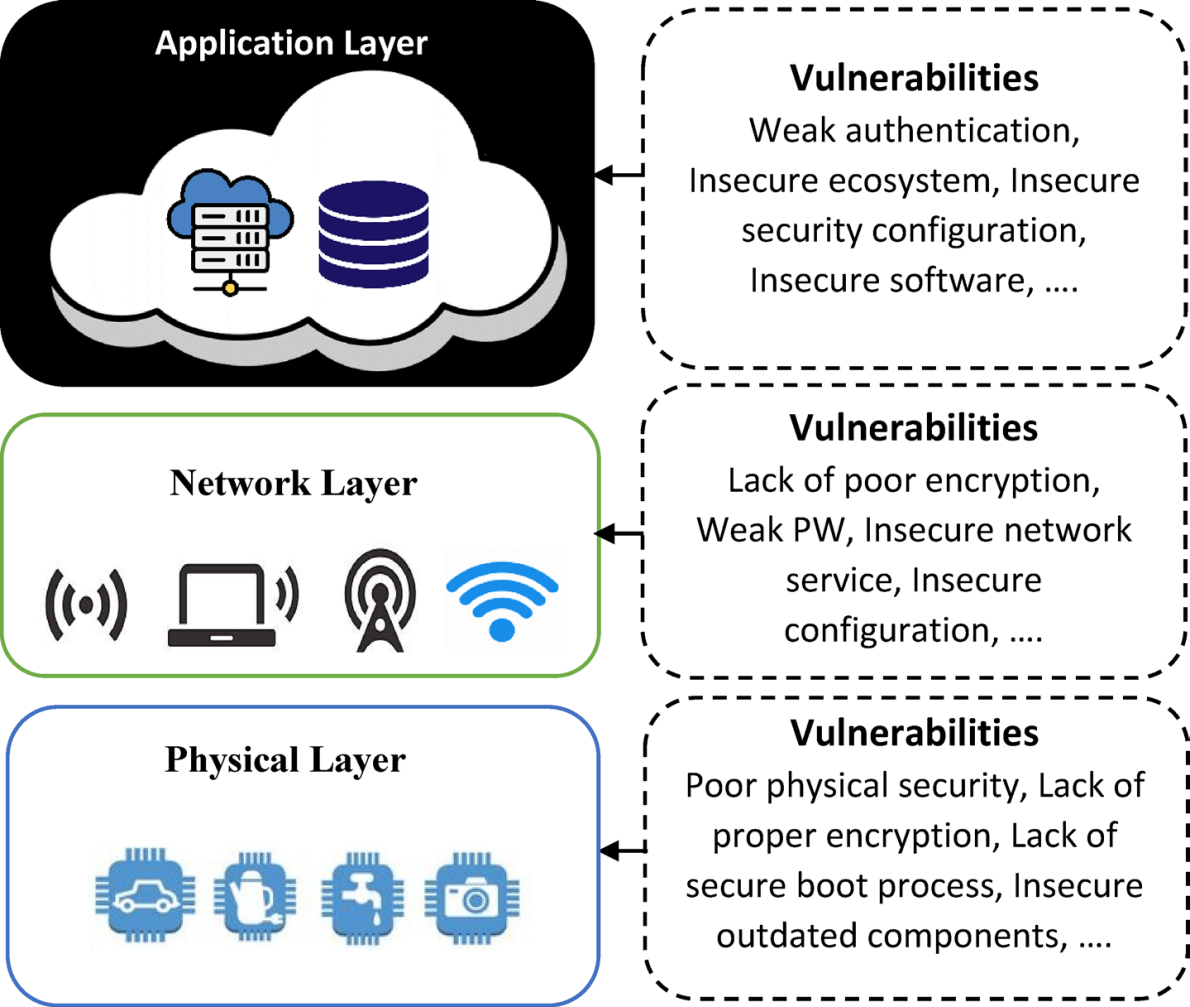
Integration of Technologies for Enhanced Airport Security.

Deep learning algorithms are adept at pattern recognition and complex data analysis because they are based on the structure and functions of the human brain. As a result, they can be used to identify irregularities in sensor data and video recordings, which could reveal suspicious activity or abandoned objects. The integration of deep learning algorithms, intelligent recommendation systems, and Internet of Things devices in the suggested architecture to enhance airport security is shown in Fig. 1.

Researchers are developing innovative frameworks that could transform airport security by fusing these promising technologies with intelligent recommendation systems. By dynamically assigning staff and resources to high-risk regions, these frameworks can optimize security procedures by utilizing historical trends and real-time data. Intelligent recommendation systems are also capable of ongoing learning and adaptation, which enhances their capacity to identify irregularities and lessen risks over time^3^.

Given the rising frequency of air travel and the evolving nature of potential threats, it is imperative that airports maintain strict security measures in the modern environment. Conventional security systems sometimes struggle to accurately identify suspicious activity and conduct effective surveillance over large areas. This necessitates researching innovative techniques that make use of cutting-edge technologies. New developments in deep learning algorithms and Internet-of-things (IoT) devices provide encouraging prospects for enhancing airport security. Through the integration of these technologies with intelligent recommendation systems, researchers are putting forth novel frameworks that have the potential to significantly improve existing security measures.

Airport security could benefit from advanced recommendation systems, but existing versions have limitations. Accurate classification and analysis are difficult due to the intrinsic complexity of airport environments, which are marked by a wide range of activities and behaviors. Additionally, some algorithms might not fully integrate real-time data into their suggestions and struggle to adjust to new risks. The effectiveness of security might also be hampered by false alarms and inefficient resource allocation. The development of more robust and adaptable recommendation algorithms that effectively manage the intricacies of airport security is necessary to address these issues^6^.

To improve security standards, this study looks at a framework that uses IoT-enabled cameras combined with deep learning for anomaly detection and intelligent recommendation systems. The suggested method aims to decrease false alerts, increase operational efficiency, and improve security. Through case studies and simulations, the effectiveness of this framework is evaluated, demonstrating its potential to improve airport security.

The combination of IoT sensors, deep learning algorithms, and intelligent recommendation systems is causing a paradigm shift in airport security. The shortcomings of current systems are addressed by this all-encompassing approach. By using the IoT devices’ capacity to gather and evaluate data in real-time, airports can obtain important insights on passenger behavior, luggage handling, and general terminal operations. Deep learning algorithms that effectively and precisely analyze data to spot anomalies, including unattended bags or suspect activity, improve this capability.

This study is then advanced by intelligent recommendation systems, which dynamically improve security tactics based on known threats and previous patterns. By directing resources to regions that are most in need, this proactive approach improves operational efficiency and security. One of the main advantages of this integrated framework is its capacity to reduce false alarms, a prevalent problem with traditional security systems. By integrating real-time data analysis with historical trends, the system distinguishes between legitimate security risks and benign activity, lowering the possibility of needless disruptions. Furthermore, intelligent recommendation systems’ insights-driven dynamic resource allocation guarantees the appropriate deployment of security personnel, improving the overall efficacy of security operations.

In addition to addressing pressing issues with airport security and laying the groundwork for future developments in the field, this creative solution ensures the safety and security of airports in a constantly changing environment.

Problem statement

Airport security measures must be improved due to the growing volume and complexity of air travel activities. Potential security flaws may result from existing systems’ inability to effectively monitor vast, dynamic environments and adjust to the changing nature of attacks. By creating a novel framework that combines the strength of intelligent recommendation systems for optimal security protocols with IoT-enabled deep learning for anomaly detection, our research seeks to overcome these constraints.

Motivation of the study: the increasing scale and complexity of modern airports pose significant challenges to traditional security systems, which often rely on manual monitoring, static rules, and isolated detection technologies. These approaches struggle to handle real-time, multi-source data and frequently result in high false-alarm rates and inefficient resource utilization. Motivated by the lack of integrated solutions that jointly address threat detection and operational decision-making, this study proposes an IoT and deep learning–based intelligent security optimization framework. The proposed ISODI framework unifies real-time sensing, advanced anomaly detection, and adaptive resource allocation to enable proactive, efficient, and risk-aware airport security management.

Novelty of the proposed framework: although Multi-View Convolutional Neural Networks (MVCNN) were initially proposed in 2015 for 3D shape recognition tasks^6^, their application has remained largely limited to visual object classification and related domains. In contrast, this study introduces a novel application of the MVCNN technique in the domain of airport security, marking the first known attempt to harness its capabilities for real-time anomaly detection using IoT sensor data.

The proposed ISODI framework uniquely integrates MVCNN within a broader system that also includes CNNs, RNNs, intelligent recommendation modules, and dynamic security protocol optimization. By combining transfer learning with real-time inputs from multiple airport systems (e.g., surveillance cameras, sensors, access control), ISODI effectively addresses the critical challenge of timely and accurate threat prediction in security-sensitive environments. This integration and domain-specific deployment represent a significant advancement beyond the original scope of MVCNN, thereby ensuring the novelty of our contribution.

Main contributions in this paper can be summarized as follow:

- Proposed ISODI framework: presented an intelligent anomaly detection framework driven by the Internet of Things that combines real-time data and deep learning to improve airport security.
- Hybrid deep learning approach: combined spatial and temporal feature extraction, CNNs and RNNs were used to detect anomalies accurately.
- Integration of transfer learning: to enhance performance on limited and domain-specific datasets, pre-trained models and transfer learning (MVCNN) were utilized.
- Optimization of security protocols: a dynamic resource allocation technique that adapts security protocols in real time according to risk assessment was developed.
- Continuous adaptation mechanism: real-time feedback and data streams were used to implement an adaptive phase for continuous learning and system changes.
- Performance evaluation: outperformed current techniques with better results (F1-score: 0.99, Precision & Recall: 1.0), demonstrating ISODI’s ability to eliminate false positives and identify all real anomalies.

Here is how the remaining work is arranged: An overview of the literature is provided in section "Literature Review". The suggested approach is covered in Section "IoT and Deep Learning-Based Intelligent Security Optimization (ISODI)". Section "Conclusion" provides an experimental evaluation. Finally, we wrap off this task in section "Future Work:".

## Literature review

Security control in the airports should be improving continuously to meet the changing threats due to the fast development of international air travel^1^. Traditional security systems are usually unable to efficiently patrol massive airport terminals and avert suspicious activities instantly because they are overly dependent on human oversight and the inactive infrastructure^1^. These restrictions may jeopardize the security of passengers, staff and valuable airport facilities. The new technological developments provide a good hope on such issues. Internet of Things (IoT) technologies can be used to allow airports to install interconnected sensors and surveillance systems, which will give real-time and continuous data on the movement of passengers, baggage handling, and operational conditions^2^. Nevertheless, IoT-generated data is vast and heterogeneous, which requires smart analysis techniques to be able to draw actionable information.

Deep learning methods have proven to be particularly effective in handling high-dimensional information that is complex and detecting minor patterns in highly varied data modalities^3^. Deep learning models can be useful in identifying abnormal behaviors, unattended objects and possible security threats by processing sensor streams and surveillance photos. Although the existing literature has already researched deep learning in terms of detecting threats and offering recommendations on resource allocation separately, the literature has not paid much attention to this in terms of integrating both into a single framework. This deficiency in end-to-end integration inhibits the versatility and effectiveness of the existing security systems of the airport, which underscores the necessity to have smart architectures that collectively exploit real-time IoT sensors, deep learning-based anomaly detection, and adaptive security decision making.

Implementing comprehensive security checks at airports is a critical component of guaranteeing the safety of air travel. The newest technology used is the High Definition-Advanced Imaging Technology (HD-AIT) system, which creates high-quality 3D full-body scans using millimetres wave scanners to handle the changing threats and improve transit security. However, there is a need for more precise prediction techniques because the existing autonomous assessment of possible dangers from scan pictures frequently produces large percentages of false alarms^4,5^.

Utilizing the potential of 3D images is a major problem in the field of computer vision research. For object categorization on 3D surfaces, some research supports volumetric Convolutional Neural Network (CNN) designs; nevertheless, these methods are computationally demanding. As an alternative, training on several 2D views has become a practical strategy with better performance and outcomes. In order to improve threat detection accuracy, this study uses multi-view CNN (MVCNN) to project 2D images at different angles from TSA’s 3D full-body scans^6^, ^7^.

Su et al.^6^ introduced the Multi-View Convolutional Neural Network (MVCNN) for the recognition and classification of 3D shapes using rendered views on grayscale 2D images. Their architecture incorporates a pre-trained CNN from ImageNet^8^ and integrates multiple views of an object. The approach utilizes multi-view pooling, which combines all views through element-wise maximum pooling to synthesize information. The output from this pooling operation serves as input to a second CNN, producing the final output. LeCun et al.^9^ proposed a similar methodology for stereo images. Su et al.^6^ conducted experiments to determine the optimal number of views, finding that 80 views yield similar accuracy to 12 views. Qi et al.^5^ enhanced MVCNN performance through data augmentation, including azimuth and elevation rotations.

Using a somewhat modified architecture, Geras et al.^10^ used the MVCNN technique to high-resolution medical pictures. After concatenating many views into a fully linked layer, they distributed the output using a SoftMax layer. We adopted modified versions of Su et al.^6^ and Geras et al.^10^ techniques to detect threats from full-body scans since we recognized the applicability of MVCNN in a variety of disciplines.

The usefulness of computer-based training (CBT) for security screeners’ interpretation of X-ray images has been investigated in several studies (e.g.,^14–16^). X-Ray Tutor (XRT) is a well-known illustration of this type of instruction^16,17^. Screeners are shown hundreds of different X-ray images of passenger baggage filled with different forbidden objects during an XRT session. By considering various rotations, levels of superposition, and bag complexity, this training enables students to understand how these forbidden objects appear within X-ray images^17^. An essential component of effective learning is the program’s instant feedback on how accurate their answers were^17,18^.

According to research, XRT training can speed up the identification of hazardous objects in passenger luggage and greatly enhance detection performance^19^. Notably, screeners frequently take longer to analyse images that lack target objects, or “target absent” images^20,21^. Maintaining effective passenger flow requires avoiding false alarms, achieving quick response times, and taking other considerations such the availability of explosive detection systems (EDS) into account^22^. A comparison of algorithms used in earlier studies for danger identification in airport security is shown in Table 1. It lists the descriptions, benefits, and drawbacks of the algorithms according to the corresponding references.

**Table 1:** Evaluation of earlier algorithms.

Algorithm	Reference	Description	Pros	Cons
3D CNN	^1^	Volumetric CNNs designed for object classification on 3D surfaces	Effective for 3D object classification	Computationally expensive training on 3D images
MVCNN	^6^	Multi-View Convolutional Neural Network for recognizing and classifying 3D shapes using rendered views on 2D grayscale images	Utilizes multiple object views for improved accuracy	Rely on pre-trained CNNs from ImageNet
MVCNN with data augmentation	^5^	Enhanced MVCNN performance through data augmentation techniques, including azimuth and elevation rotations	Improves accuracy by increasing data variability	Augmentation process may introduce noise or artifacts
MVCNN for medical images	^10^	Adapted MVCNN architecture for analyzing high-resolution medical images	Versatile approach applicable to various domains	May require architectural modifications for specific applications

The following are some of the main drawbacks of earlier threat detection algorithms for airport security:

- Computational intensity: training 3D CNNs necessitates a substantial amount of processing power and numerous training samples, which could not be practical in resource-constrained real-world situations.
- The dataset imbalance, in which about 80% of the data is classified as “threat” and 20% as “no threat,” might result in biased models and less-than-ideal performance.
- Managing 3D images: because 3D images are complicated and require specific hardware and algorithms, processing and training them can be difficult.
- False alarms: a common problem with current systems, false alarms can lower productivity and raise operating expenses.
- Limited integration: deep learning and recommendation algorithms are not integrated into a single framework for all-encompassing airport security, which reduces the system’s overall efficacy.

## Areas requiring further research:

- Lack of integrated frameworks: existing research often examines deep learning for threat detection and recommendation systems for resource allocation separately. The integration of these technologies within a unified framework for comprehensive airport security remains under-explored.
- Handling complex airport dynamics: current recommendation systems may not effectively address the diverse range of activities, passenger behaviors, and constantly evolving threats within complex airport environments. This can lead to inaccurate analysis and inefficient allocation of security resources^11^.
- Mitigating false alarms and optimizing resource allocation: current security systems frequently encounter challenges related to false alarms and inefficient resource allocation. Further research is needed to investigate how integrating intelligent recommendation systems with real-time data analysis can effectively address these limitations^12,13^.

Existing airport security studies rely on isolated detection models, computationally expensive 3D CNNs, and static decision rules, often leading to high false-alarm rates and limited real-time adaptability. The proposed ISODI framework overcomes these limitations by integrating IoT-driven sensing with computationally efficient MVCNN-based deep learning and an intelligent recommendation system that dynamically optimizes security resources based on real-time risk assessment.

## IoT and deep learning-based intelligent security optimization (ISODI)

**Fig. 2:**
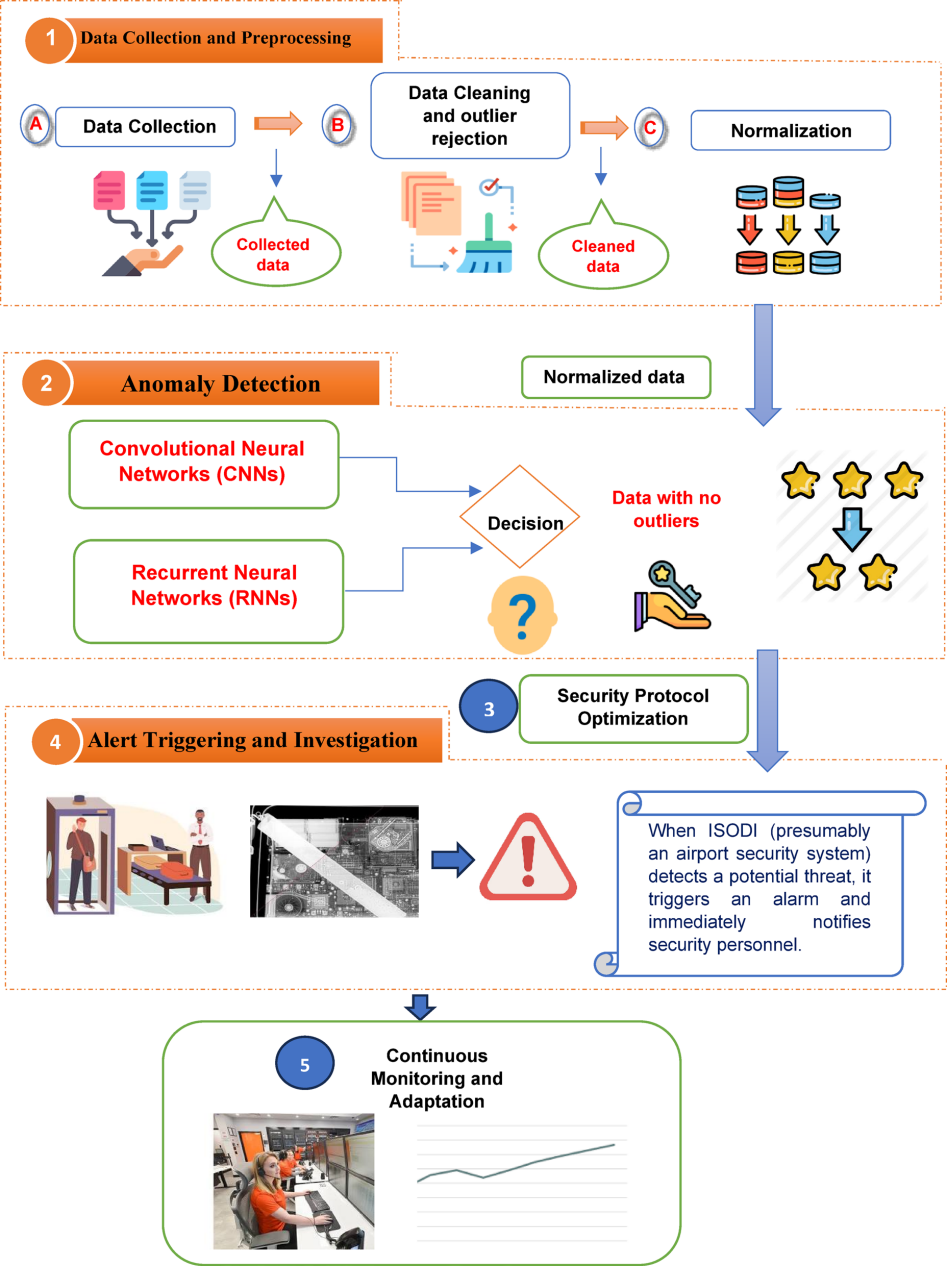
Intelligent security optimization through deep learning and IoT (ISODI) framework.

IoT and Deep Learning-Based Intelligent Security Optimization (ISODI) is a new framework that uses Internet of Things (IoT) and deep learning technology to transform airport security as depicted in Fig. 2. This all-encompassing strategy incorporates real-time data from multiple sources, such as airport systems, sensors, and surveillance cameras.

## ISODI operates in five key stages:

- Data acquisition and preparation: in the initial stage, real-time data is collected from various sources and prepared for analysis. This includes handling missing data, standardizing features, and ensuring data compatibility with the subsequent analysis algorithms.
- Anomaly detection using deep learning: advanced deep learning models, such as Convolutional Neural Networks (CNNs) and Recurrent Neural Networks (RNNs), are employed to analyse the pre-processed data.
- Security protocol optimization: based on the insights gained from anomaly detection, intelligent recommendation systems are utilized to dynamically optimize security protocols. This involves real-time allocation of resources and personnel based on assessed risks and predicted threats, enabling proactive security measures.
- Alert generation and response: When potential threats are identified, ISODI triggers alerts and notifies security personnel for immediate investigation. This ensures prompt response to security incidents, minimizing risks to passengers and airport infrastructure.
- Continuous monitoring and improvement: ISODI continuously monitors security operations by gathering feedback and adapting its algorithms based on real-time data and input from security personnel. This adaptability ensures the system remains effective against evolving threats and maintains optimal efficiency over time.

By integrating these functionalities, ISODI aims to significantly enhance airport security standards, streamline operational efficiency, and contribute to a safer environment for both passengers and airport staff.

### Data collection and preprocessing

The ISODI system combines the heterogeneous IoT sensors that will be installed throughout the airport facilities to allow real-time and full security coverage. These sensors consist of the IoT-enabled surveillance cameras (high-definition CCTV cameras and millimetre-wave cameras that scan the entire body), motion sensors and proximity sensors to identify the abnormal movement patterns, access control sensors (RFID reader and badge-based authentication) and environment sensors to measure the operational conditions of restricted zones. The data of such sensors are inter-temporally synchronized and sent to a single layer of data acquisition, where preprocessing, normalization, and feature alignment can be done. The merged multimodal data are subsequently sent to the anomaly detection module based on the deep learning mechanisms to enable the system study visual, spatial, temporal, and contextual data together in a bid to effectively identify threats and optimize security.

ISODI gathers real-time data from a range of airport-related sources, such as sensors, security cameras, and airport systems, during the “Data Collection and Preprocessing” stage. Preprocessing is the following step in guaranteeing the caliber and suitability of this data for the forthcoming analysis processes. Preprocessing prepares the data for entry into the deep learning algorithms that identify abnormalities by handling missing values and standardizing characteristics. The main steps of the Data Collection and Preprocessing Algorithm (DCPA) are described in Algorithm 1.

**Algorithm 1:**
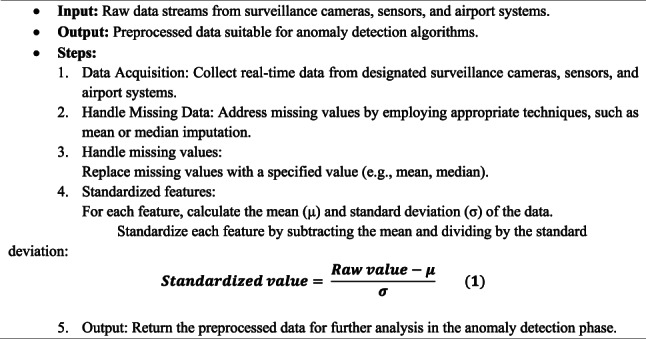
Data Collection and Preprocessing Algorithm (DCPA)

This method guarantees that the information gathered is standardized, clean, and prepared for examination in later stages of the ISODI framework:

- Stressing data quality: By standardizing, cleaning, and getting the data ready for additional analysis within the ISODI framework, this procedure ensures the quality of the data that has been gathered.
- Putting an emphasis on analysis readiness: The algorithm cleans up the data and makes sure it works with the analysis methods used later in the ISODI framework.
- Emphasizing the algorithm’s function: This stage is essential to converting unprocessed data into a format that can be used for analysis in the ISODI framework’s later phases.
- A more straightforward option: The algorithm cleans and gets the data ready for analysis in the ISODI framework’s later sections.

Before detecting the anomalies, the IoT sensor data and surveillance videotapes are first thorough preprocessed through a pipeline process that guarantees the data reliability and the data analytics process is robust. Sensor stream Missing values within sensor streams are corrected by applying statistical imputation algorithms based on the distribution of data and the sensor type (mean or median). Temporal smoothing and filtering methods are used to eliminate noise and signal variations inherent in the measurement process of the IoT to maintain spurious variations and relevant patterns.

In the case of camera footage, preprocessing involves frame resizing, intensity normalization and denoising to normalize image quality and minimize visual artifacts. Applicable frames are obtained at uniform time rates to strike a balance between computation efficiency and time coverage. The CNN-based models are then used to extract features based on spatial data of image data and the temporal relationships and sequencing patterns in sensor streams are represented by RNN-based structures. Such an integrated preprocessing approach can guarantee the transformation of multimodal information across heterogeneous IoT sources into standard and quality representations that can be subjected to the deep learning-based anomaly detection in the ISODI framework.

### Anomaly detection with deep learning

In the “Anomaly Detection with Deep Learning” phase, ISODI uses advanced deep learning methods such as Convolutional Neural Networks (CNNs) and Recurrent Neural Networks (RNNs) to examine the preprocessed data and identify anomalies. Because these algorithms are trained on labeled data to recognize trends and deviations from normal behavior, the sensor data and camera footage can be used to detect suspicious activity, unattended luggage, and other potential security issues. The main steps of the Anomaly Detection with Deep Learning Algorithm (ADDA) are described in Algorithm 2.

**Algorithm 2:**
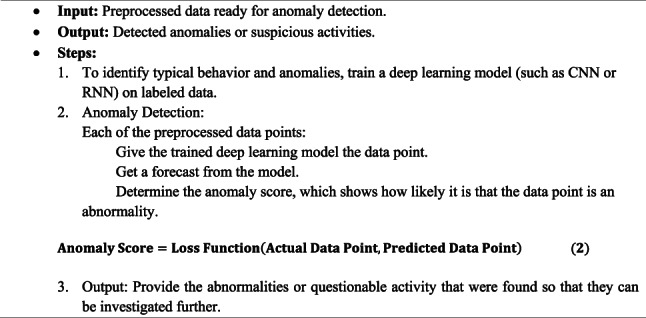
Anomaly Detection with Deep Learning Algorithm (ADDA)

The deployment of proactive steps to ensure the safety of passengers and employees is made possible by this algorithm, which gives ISODI the ability to actively identify abnormalities and potential security threats in the airport environment. Both a lot of examples and a lot of processing power are needed to train a full 3D CNN. Since our dataset of 1,147 three-dimensional complete body scans is insufficient to fully train a new 3D CNN, we choose to employ MVCNN rather than train for full 3D CNN. Another method for classifying or predicting 3D objects is MVCNN, which uses 2D photos taken from different perspectives^6,7^. Utilizing pre-trained 2D CNNs that are available on ImageNet databases is another benefit of downsizing from 3 to 2D^8^. Using a pre-trained VGG16 on the ImageNet^8^, we created two different kinds of MVCNNs in this study: (i) Fine-tuning MVCNN and (ii) Transfer learning MVCNN.

Data and computational constraints have forced the use of transfer learning with MVCNN architecture, as opposed to making a fresh start with a new model. The set of passenger screening data they have has few labeled 3D body scans, and this is not enough to effectively train deep volumetric or multi-view networks without the high probability of overfitting. Full 3D CNN training would be computationally expensive and ensure long training time as well, which would not be practical to apply in a real-time airport security scenario. Using a VGG-16 backbone that has been trained on the ImageNet dataset, the proposed MVCNN utilizes rich and general-purpose visual features that were trained on large datasets. This method offers a major benefit of minimizing the complexity of training, increasing convergence speed, and generalization performance, with a high detection rate. Therefore, transfer learning-based MVCNN is a computationally efficient and deployable solution highly appropriate to the environment of airport security.

i. Fine-tuning MVCNN

**Fig. 3:**
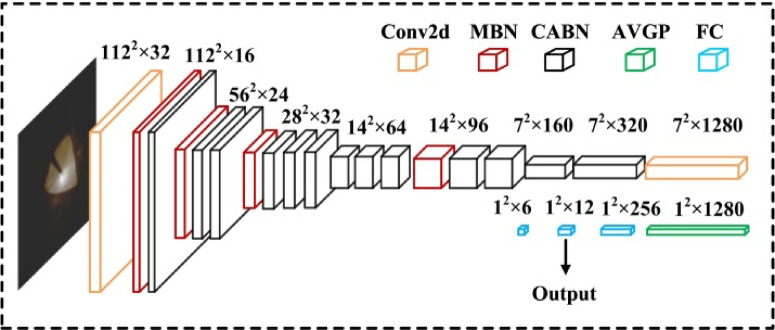
VGG-16 architecture.

**Fig. 4:**
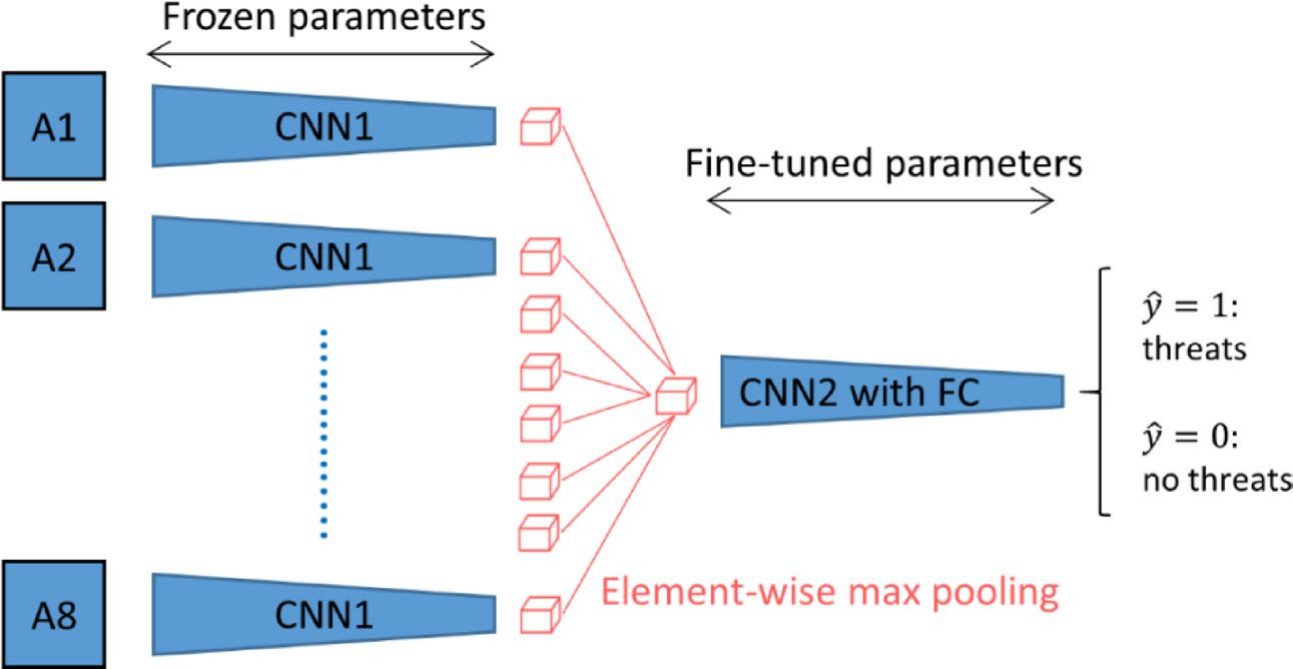
The connection between CNN1 and CNN2 with fully connected layers.

As seen in Fig. 3, we divided the VGG-16 design into two halves, which we call CNN1 and CNN2. CNN2 begins at the last conv2D group to the feature extraction layer, while CNN1 begins at the input to the final conv2D group of the VGG-16 model. By training the CNN2 and fully connected layers on our training set, we improved their performance. The kernel and bias parameters from the first portion of the pre-trained VGG-16 on the ImageNet were applied to the CNN1 section. As illustrated in Fig. 4, each 2D body scan from an angle was subjected to the forward propagation of the pre-trained CNN1, yielding an extracted feature cube with dimensions of 14 × 14 × 512. CNN1’s outputs from all eight-angle inputs are combined using the element-wise max pooling operation for multi-view pooling (Fig. 4). CNN2 and fully connected layers now accept the pooled feature layer as an input. During the training phase, CNN2’s fully connected layer parameters are adjusted.

CNN2 (the final Conv2D group of VGG-16) and CNN1 (the first section of VGG-16) were designed with completely connected layers. Using our dataset, we optimized CNN2’s second section with completely connected layers. Figure 4 illustrates the completely connected layer relationship between CNN1 and CNN2. With completely connected layers, CNN1 and CNN2 are joined via the element-wise maximum pooling method.

ii. Transfer learning MVCNN

**Fig. 5:**
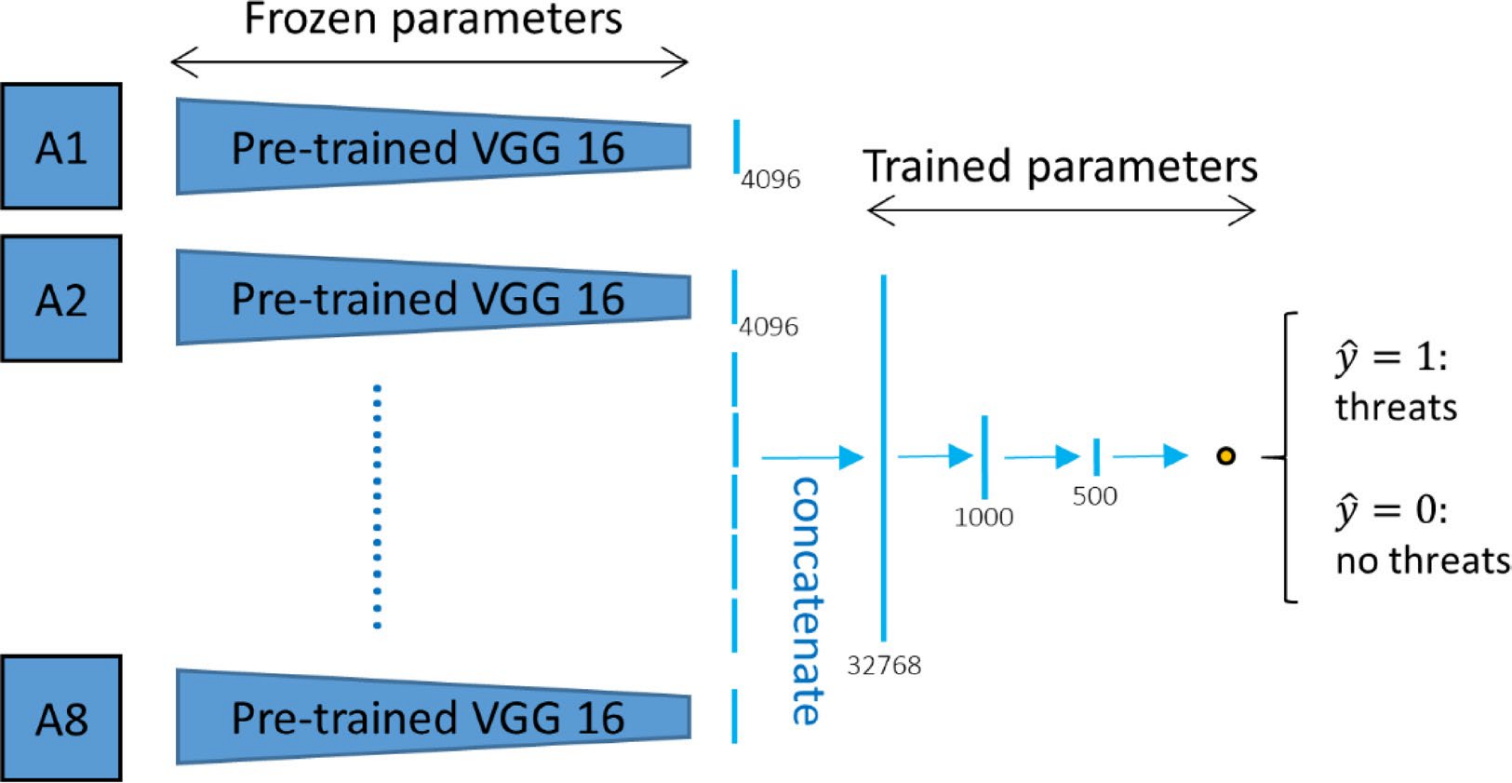
Transfer learning with MVCNN.

Unlike the fine-tuning MVCNN, we forward propagated the input image and obtained the feature extraction output by using the kernel and bias parameters from the pre-trained VGG-16 model on the ImageNet, as illustrated in Fig. 5. The pre-trained VGG-16 was applied to each input image at an angle, and the features were extracted to a vector with a length of 4096. Concatenating the characteristics and running them through completely connected layers allowed us to merge all the photos taken from various perspectives. Logistic regression is used to get the output prediction (Fig. 5). Pre-trained VGG16 is used to extract each image’s features into a 4096-length vector. For logistic regression, the characteristics from every perspective are concatenated and run through the fully connected layers.

### Security protocol optimization

ISODI uses information from anomaly detection to optimize security protocols in real-time during the “Security Protocol Optimization” step. Intelligent recommendation systems analyze the data to assess the risks associated with the abnormalities discovered. In response to these evaluations, security procedures are adjusted dynamically to allocate staff and resources efficiently, focusing on high-risk and possibly hazardous areas. Algorithm 3 provides a thorough explanation of each phase of the Security Protocol Optimization Algorithm (SPOA).

**Algorithm 3:**
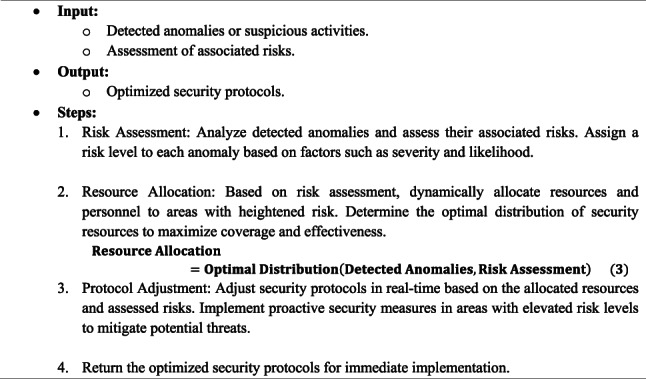
Security Protocol Optimization Algorithm (SPOA)

To constantly improve security protocols and make sure that security measures properly address the unique dangers that exist within the airport, ISODI makes use of real-time data and risk assessments. This data-driven strategy reduces possible threats and greatly increases overall security efficacy.

The MVCNN model used in ISODI is developed on the VGG-16 network that is pre-trained on ImageNet. Every 3D whole body scan is described by several 2D projections taken by eight different perspectives. All the projections are handled individually with the help of the common-weight CNN backbone. The elements of the final convolutional layer (14*14*512) are summarized through element-wise max pooling in order to create one unified multi-view representation, namely the feature maps. This shared representation is then passed on fully connected layers to make a classification.

Two types of MVCNNs are adopted, namely: fine-tuning MVCNN and transfer learning MVCNN. In the fine-tuning setup, initial convolutional layers are fixed and subsequent layers and fully connected layers are added and trained on the airport security data at a learning rate of 1*10^–4^.

A batch size of 16 and early stopping to avoid overfit the system. VGG-16 backbone in the transfer learning setup is applied as a fixed feature extractor only, generating 4096 dimensional feature vectors per view. These vectors are then concatenated and run through fully connected layers and finally threat classification is done using logistic regression. The specifics of the CNN, RNN and MVCNN architectures and the respective hyper parameters employed in the ISODI framework are outlined in Table 2.

**Table 2:** Deep learning architecture and hyperparameters used in ISODI.

Model	Key layers	Hyperparameters
CNN	Conv(3 × 3) + ReLU, MaxPool, Dropout, FC	LR = 1e-4, Adam, Batch = 16
RNN (GRU)	GRU layers, FC	Time window-based input
MVCNN	VGG-16 backbone, max view-pooling, FC	Views = 8, LR = 1e-4, Early stopping

### Alert triggering and investigation

ISODI alerts security experts and starts the “Alert Triggering and Investigation” step when it discovers potential threats or anomalies. After receiving an alarm, security personnel promptly start an investigation and response protocols to lessen risks to passengers and airport property. At this point, prompt action is ensured, and security-related problems have less of an impact on airport operations. Algorithm 4 provides a detailed example of the Alert Triggering and Investigation Algorithm (ATIA).

**Algorithm 4:**
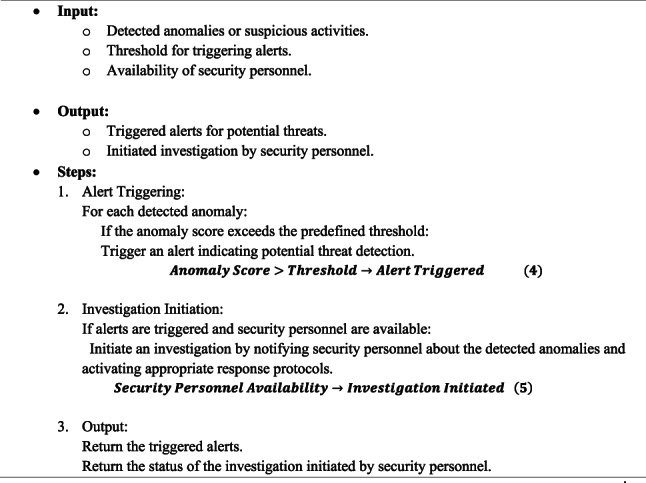
Alert Triggering and Investigation Algorithm (ATIA)

ATIA is essential for reducing security threats. This algorithm sends out alerts for possible dangers, which motivate security staff to investigate them right away. This guarantees a prompt reaction, reducing the possible impact of security events on airport operations and protecting the welfare of travelers and airport infrastructure.” (Aims to reduce the impact of security events).

### Continuous monitoring and adaptation

In the “Continuous Monitoring and Adaptation” phase, ISODI monitors security operations, gathers feedback, and adjusts its algorithms based on observations from security professionals and real-time data. This adaptability ensures that the system will remain effective against evolving threats and maintain its efficiency over time. ISODI can improve security procedures in airport environments and foresee emerging security threats by using continuous observation and adjustment. Algorithm 5 describes the main steps of the Continuous Monitoring and Adaptation Algorithm (CMAA).

**Algorithm 5:**
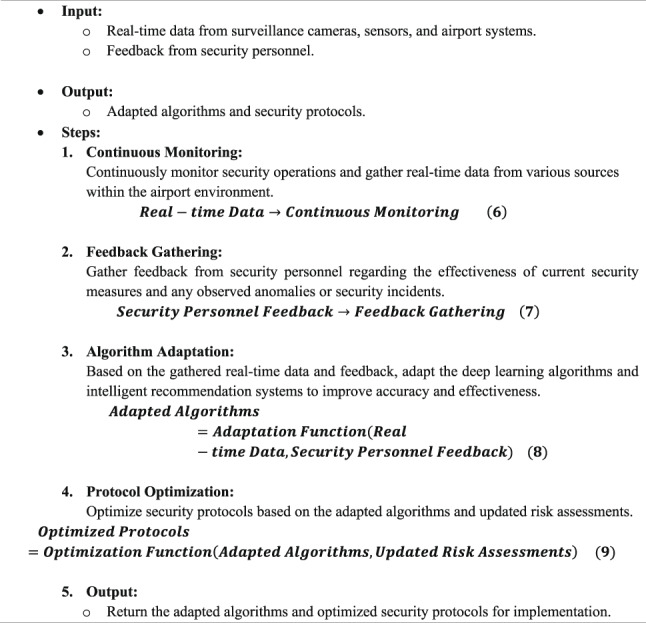
Continuous Monitoring and Adaptation Algorithm (CMAA)

ISODI protects itself from changing threats by using the Continuous Monitoring and Adaptation Algorithm (CMAA). This algorithm collects feedback, keeps an eye on security activities, and modifies its algorithms and security protocols as necessary. This proactive strategy guarantees ISODI’s continued high efficiency and ongoing enhancement of airport security measures.

## Implementation and evaluation

The performance indicators, experimental outcomes, and utilized datasets are covered in this section. Two datasets were used for analysis in this study. The first one was taken from the threat prediction competition on Kaggle and was called “Passenger Screening”^23^. More than 1,100 volunteers’ 3D scans taken from different perspectives are included in this dataset. Researchers employed 2D pictures that were flattened from these scans for computing performance. The performance of airlines is the subject of the second dataset, “Airline on-time Data^26^. It contains comprehensive flight information, including scheduled and real times, cancellations, delays, and the causes of any interruptions (weather, security, etc.).

### Passenger screening dataset

**Fig. 6:**
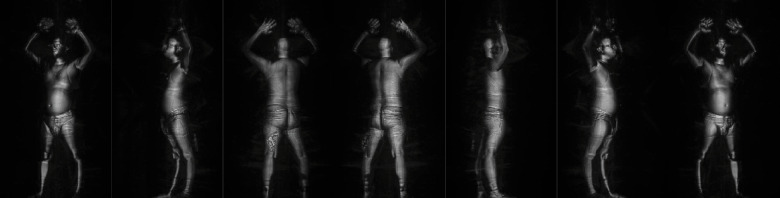
Eight grayscale images (660 × 512 pixels) generated from different angles around a 3D body scan.

For a threat prediction competition, the TSA makes available a dataset^23^ on Kaggle: https://www.kaggle.com/c/passenger-screening-algorithm-challenge. This data includes 3D scans of 1145 individuals with a range of body shapes and genders, dressed in summer through winter attire. There can be hidden dangers in the scans. Instead of using the original 3D scans, the researchers used flattened 2D images because they were more effective at detecting threats. Eight grayscale photos, each measuring 660 by 512 pixels and shot from various body angles, are provided for each volunteer. An illustration of these projected body scan images is provided in Fig. 6. The threat detection model is fed these eight pictures. An example of eight body scan photographs from various angles that are projected from a person’s 3D body scan is shown in Fig. 6. There is a gun on the left thigh in the third and fourth images from the left.

### Data split ratios

In this study, the dataset was partitioned into training, development (validation), and test sets using a ratio of 70:15:15. This division is a commonly accepted practice in machine learning to ensure that sufficient data is available for model training while preserving enough data to tune hyperparameters and evaluate generalization performance.

- Training set (70%): provides ample samples for the deep learning models to learn complex patterns effectively.
- Development set (15%): used to fine-tune model parameters and prevent overfitting by monitoring performance on unseen data during training.
- Test set (15%): held out for unbiased evaluation of the final model to assess its real-world applicability.

This ratio balances the need for robust training with reliable validation and testing, especially given the limited size of the datasets used. Moreover, stratified sampling was applied where applicable to maintain representative distributions of key classes across splits, further ensuring reliable performance assessment.

### Data collection and preprocessing

About 20% of the dataset is classified as “no-threat,” while the remaining 80% is classified as “threat,” which allows a person to have up to three things on their body. By including examples of both threats and non-threats in the training set, we were able to balance the dataset. Data augmentation enhances performance and decreases overfitting in an unbalanced dataset, according to earlier research^24,25^. The three augmentation techniques are as follows: randomly shifting the luminance of the image, sharpening images at random size, and randomly translating the body placement in a horizontal direction within a range of 100 pixels. We changed our grayscale projected body images into three bandwidths because the VGG-16 model uses RGB images as input. In a 70:20:10 ratio, the dataset is separated into training, validation, and test examples. Table 3 displays the number of photos in each set, including the training set’s original and enhanced data.

**Table 3:** Image dataset breakdown (training, augmented training, development & test).

	training set		Development Set	Test set
	Original images	Augmented images		
“Threat”	153	765	786	94
“No threat”	648	270	44	22
Total	1962		230	116

### Results of MVCNN on passenger screening dataset

**Fig. 7:**
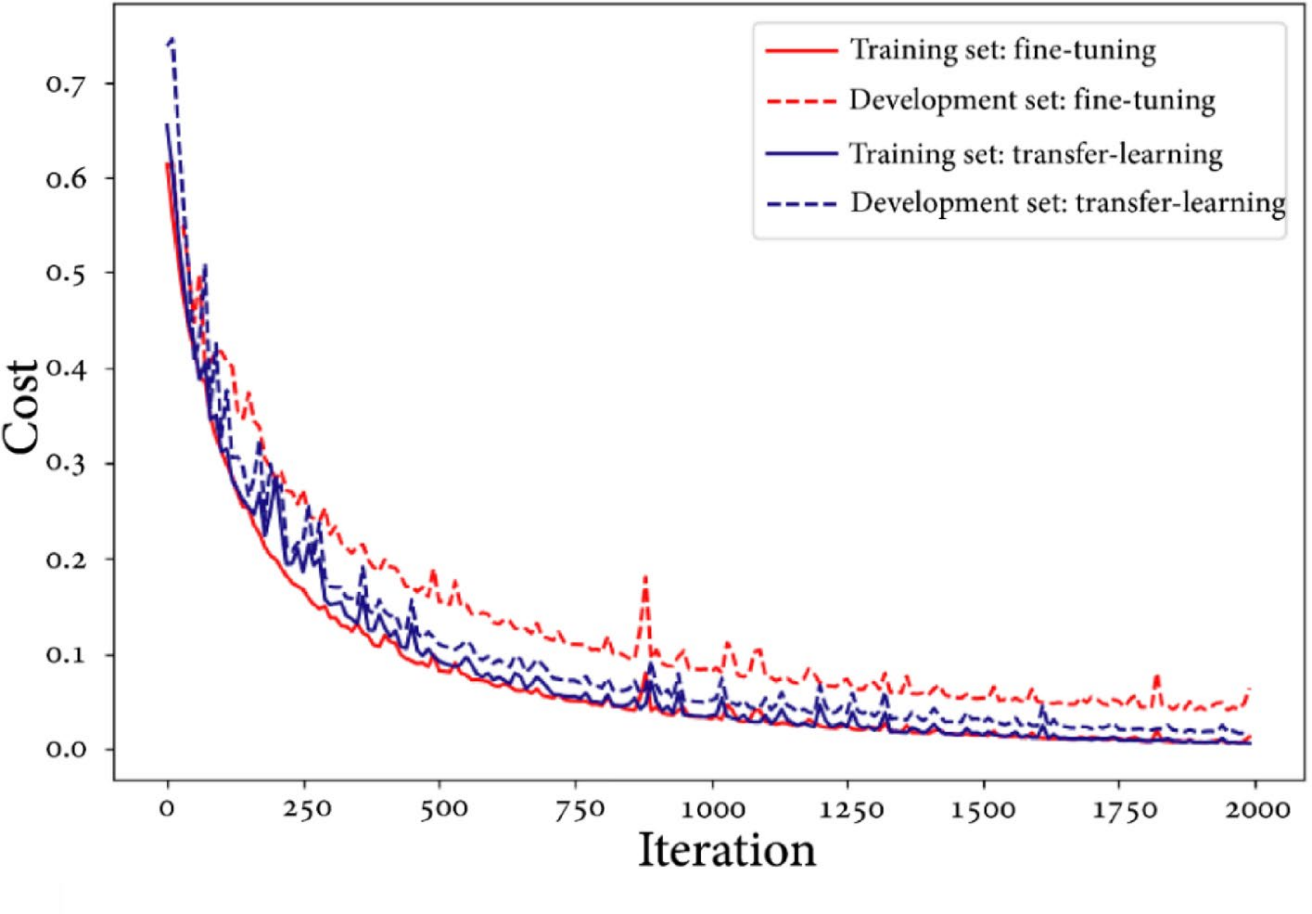
MVCNN training comparison (fine-tuning vs. transfer learning).

With above 95% accuracy in training, development, and test sets, the two MVCNN architectures provide very satisfactory overall results (Table 4). We assess our models using a variety of metrics, such as f1 scores, recall, accuracy, and precision. In every assessment technique, the transfer learning predictions outperform the fine-tuning MVCNNs. Analysis of Errors We examined the mispredictions in the test and development sets and discovered that the majority of them were caused by noisy scanned images or by individuals with unusual knee or angle appearances. Figure 7 shows the learning curves for the transfer learning (blue) and fine-tuned (red) MVCNN models. After 10 epochs, each model’s development (dashed line) and training (solid line) costs are displayed.

**Table 4:** Summary of predictions results for each type of MVCNN.

Evaluations	Fine-tuning MVCNN (no dropout) (%)	Fine-tuning MVCNN with dropout (keep_prob = 0.8) (%)	Transfer learning MVCNN (%)
Training accuracy	99.7	100	100
Dev accuracy	97.8	99.1	100
Test accuracy	95.7	94.8	99.1
Test precision	98.9	98.9	100
Test recall	95.9	94.7	99
Test f1 score	97.4	96.7	99.5

**Fig. 8:**
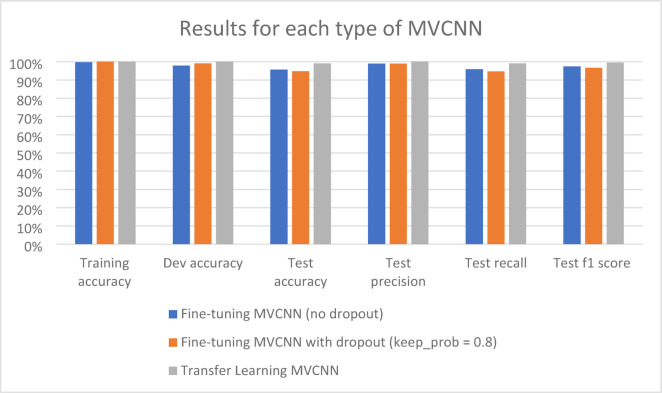
MVCNN results.

Table 3 provides a summary of the three MVCNN models’ performance. Each of the three models trained with 100% accuracy. With 100% and 99.1% accuracy on the development and test sets, respectively, the transfer learning MVCNN performed the best. On the test data, the transfer learning model showed better precision (100% vs. 98.9%) and recall (99% vs. 95.9%), even though the fine-tuned MVCNN with dropout was able to reach test accuracy (94.8%) that was comparable to the fine-tuned model without dropout (95.7%). In comparison to the fine-tuned models (97.4% and 96.7%), this results in a better F1 score (99.5%) for the transfer learning strategy. In terms of danger prediction, the transfer learning MVCNN approach produced the most encouraging outcomes overall. High training accuracy was attained by the MVCNN models, as illustrated in Fig. 8.

## Addressing overfitting and model validation

To mitigate concerns related to potential overfitting due to the high accuracy observed on the training data (Table 4), several strategies were employed. First, regularization techniques such as dropout and L2 regularization were incorporated during the model training to reduce the risk of overfitting by preventing the model from relying too heavily on any single set of features.

Second, we applied k-fold cross-validation (with k = 5) to further validate the robustness and generalizability of the model. This method involves partitioning the dataset into k subsets, iteratively training the model on k-1 folds and validating on the remaining fold. This approach ensures that the model’s performance is consistently evaluated across different data splits and not biased by a particular training/test partition.

These combined measures enhance the reliability of the reported results and confirm that the proposed ISODI framework maintains strong performance without overfitting, even when working with relatively limited datasets.

Compared to the currently reported approaches based on deep learning, the presented model composed of MVCNN is competitive and better performing, especially in the presence of transfer learning. It is indicative of how useful the suggested method is in managing small and complicated datasets.

### Results of MVCNN discussion

Three threat prediction models were examined in the study, and it was discovered that each model did well throughout training. With no errors found in a development set and an extremely low error rate (0.9%) in a separate test set, a method known as transfer learning produced the greatest performance on unseen data. This demonstrates how well the transfer learning model applies its knowledge to novel contexts. Once more, the transfer learning model performed best in terms of accuracy. In the test data, it accurately detected nearly all non-threats (99% recall) and all threats (100% precision). This corresponds to a very low rate of both missing real threats (false negatives) and misidentifying threats (false positives). According to the study’s findings, one possible method for leveraging AI to enhance threat prediction in airport security is the transfer learning methodology.

### Airline on-time dataset

The performance of airlines is monitored by the “Airline on-time Data” dataset^17^. With details grouped into features like in Table 5, it contains information on flight schedules, delays, cancellations, and diversions.

**Table 5:** Description of features in the "airline on-time performance data" dataset.

Feature	Description
ActualElapsedTime	The actual elapsed time of the flight in minutes
AirTime	The time the aircraft is in the air in minutes
ArrDelay	The arrival delay of the flight in minutes
ArrTime	The actual arrival time of the flight
CRSArrTime	The scheduled arrival time of the flight
CRSDepTime	The scheduled departure time of the flight
CRSElapsedTime	The scheduled elapsed time of the flight in minutes
CancellationCode	A code indicating the reason for flight cancellation
Cancelled	A flag indicating if the flight was cancelled (1) or not (0)
CarrierDelay	The delay, in minutes, attributed to the airline carrier
DayOfWeek	The day of the week of the flight (1 = Monday, 7 = Sunday)
DayOfMonth	The day of the month of the flight
DepDelay	The departure delay of the flight in minutes
DepTime	The actual departure time of the flight
Dest	The destination airport code
Distance	The distance traveled by the flight in miles
Diverted	A flag indicating if the flight was diverted to a different airport (1) or not (0)
FlightNum	The flight number
LateAircraftDelay	The delay, in minutes, attributed to a late-arriving aircraft
Month	The month of the flight (1 = January, 12 = December)
NASDelay	The delay, in minutes, attributed to the National Airspace System (NAS)
Origin	The origin airport code
TailNum	The aircraft tail number
TaxiIn	The taxi-in time in minutes
TaxiOut	The taxi-out time in minutes
UniqueCarrier	The unique carrier code
WeatherDelay	The delay, in minutes, attributed to weather conditions
Year	The year of the flight
SecurityDelay	The delay, in minutes, attributed to security reasons

This dataset, which is full of useful information on airline on-time performance, enables users to examine aircraft operations and analyze variables including delays, cancellations, and diversions.

### Results of airline on-time data preprocessing

**Fig. 9:**
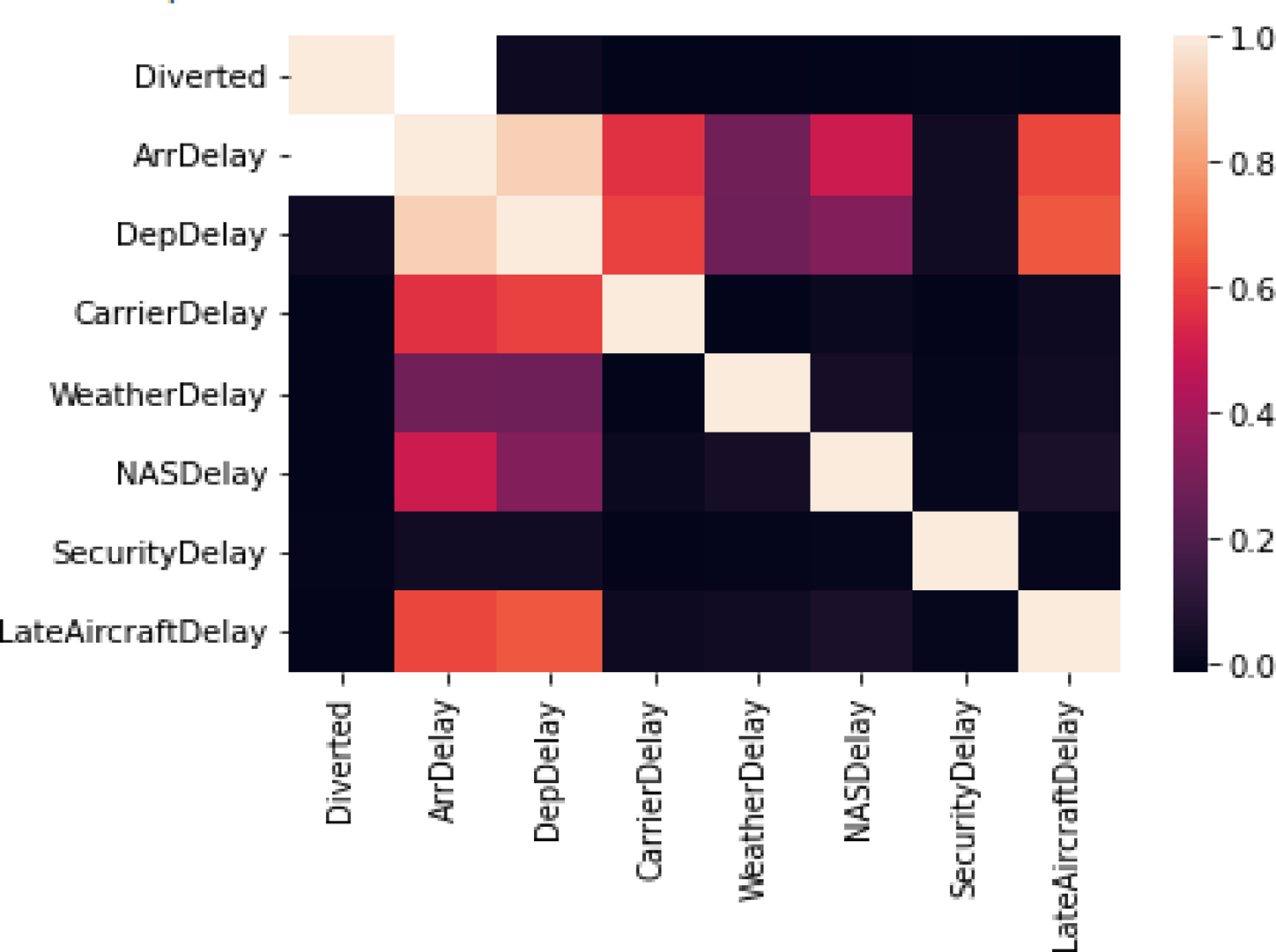
Correlation heatmap of delay and diversion variables in airline on-time data.

A heatmap analysis was performed to investigate the relationships between variables related to flight diversions (Diverted) and several delay categories (CancellationCode, ArrDelay, DepDelay, etc.) in the “Airline on-time Data” dataset, as shown in Fig. 9. Finding possible connections between these characteristics that could affect flight operations is made easier with the aid of this representation.

**Fig. 10:**
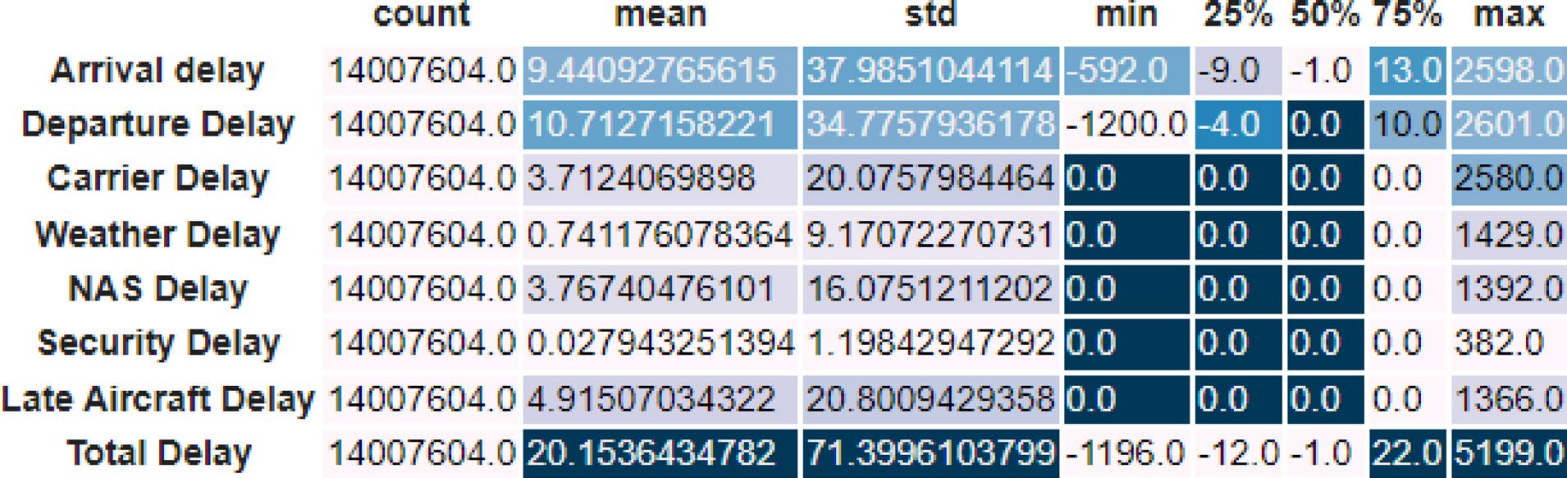
Summary statistics of delay features.

A summary table was created to provide a better understanding of the distribution of delay-related features in the “Airline on-time Data” dataset (shown in Fig. 10). The mean, standard deviation, minimum, maximum, and percentiles for each delay category (Arrival Delay, Departure Delay, etc.) and overall delay are among the descriptive data provided by this table, which was produced after choosing and renaming pertinent columns (for example, Arrival Delay to ArrDelay). Readability is improved by applying a backdrop gradient and formatting the table with a predetermined number of decimal places. Initial comprehension of the variety and extent of delays encountered in the dataset is made possible by this thorough summary.

Figure 9 presents a heatmap illustrating the correlation between key features associated with flight delays and diversions in the “Airline On-Time Data” dataset. The heatmap provides a visual overview of the relationships between variables such as ArrDelay (Arrival Delay), DepDelay (Departure Delay), CancellationCode, Diverted, and other delay-related attributes.

This visualization aids in identifying significant positive or negative correlations among variables, which may reveal underlying patterns impacting flight disruptions. For instance, strong positive correlations between DepDelay and ArrDelay suggest that departure delays are likely to lead to arrival delays. Meanwhile, the correlation between Diverted and other variables helps in understanding how delays or cancellations might influence flight diversions.

The color intensity in the heatmap represents the strength of these correlations, with darker shades indicating stronger relationships. This graphical analysis supports the exploratory data phase of ISODI, informing the selection of features most relevant to anomaly and threat detection in airport operations.

Figure 10 shows the statistical distribution with the help of the box plot, of main delay-related features. This representation, as opposed to traditional tabular summaries, gives a better idea of the distribution of the spread and the central tendency of the delay values as well as an idea of the spread of outliers. The figure indicates that the relatively small delays on most flights, and a relatively few extreme values result in long-tail distributions. This visualization is useful in the series of anomaly detection because it is used to clearly identify outlier behaviors that can be associated with abnormal operational conditions.

**Fig. 11:**
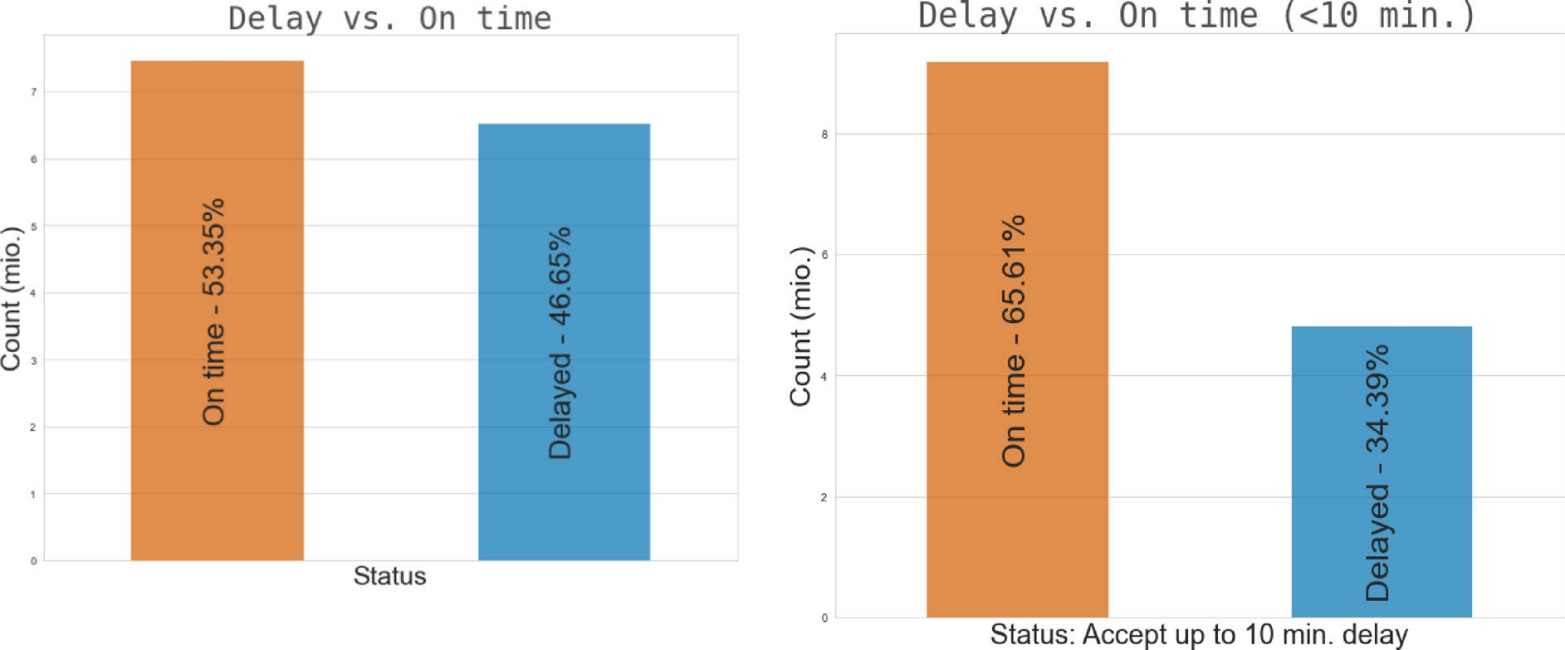
Distribution of arrival and departure delays.

Two bar charts are combined in Fig. 11 to show the distribution of delayed and on-time flights in the dataset under analysis. Both percentages and the total number of flights (in millions) are used to display the statistics. While the lower chart concentrates on flights with a maximum delay of ten minutes, the top chart considers all flights. This makes it possible to analyze flights classified as “on-time” based on a certain delay threshold in greater detail.

Figure 11 illustrates the frequency distribution of arrival and departure delays. The figure highlights the skewed nature of delays, with the majority of flights experiencing short delays but with a long tail representing more severe disruptions. The distribution insights support the anomaly detection task by emphasizing the outlier behavior of delayed flights.

**Fig. 12:**
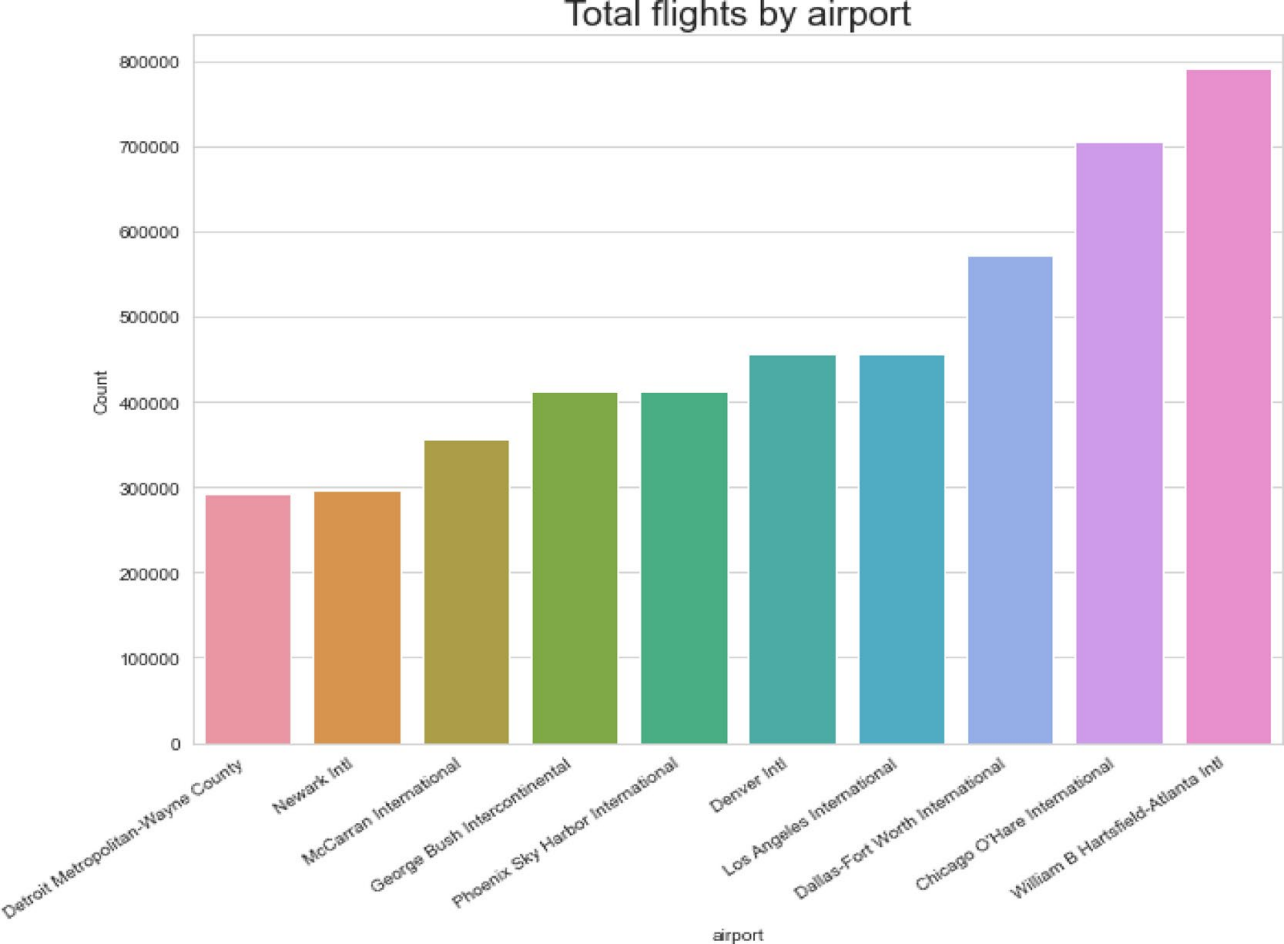
Airport-wise flight volume, delay count, and delay rate.

**Fig. 13:**
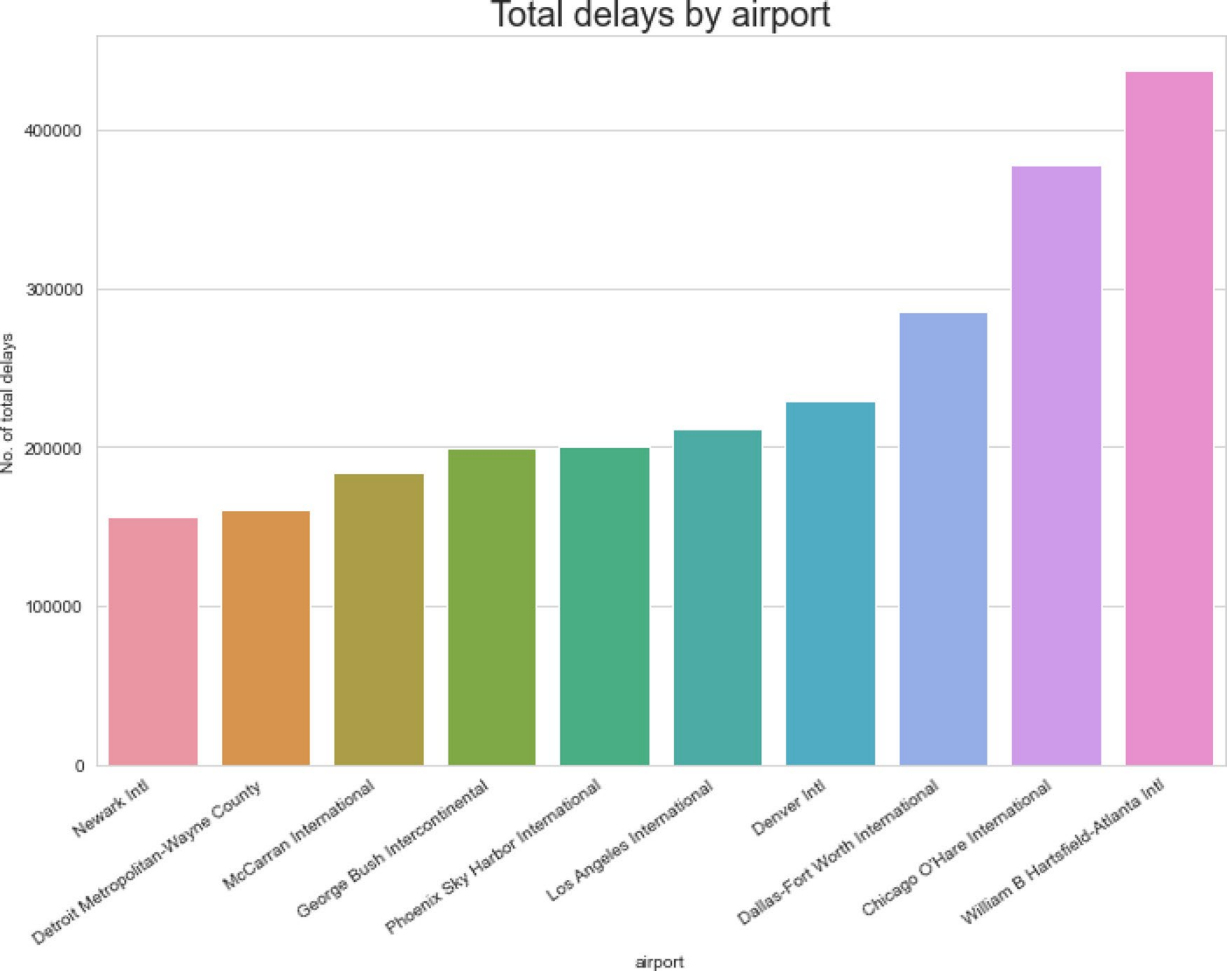
Analysis of delays by airport (airline on-time data).

Within the “Airline on-Time Data” dataset, Figs. 12 and 13 examine flight activity and delay trends at several airports. There are three different bar charts displayed. The first looks at how many flights each airport handles overall. The second investigates how many delays there were overall at each airport. The third chart, which comes last, examines the proportion of flights that experience delays in relation to all flights at each airport. Airports with perhaps higher traffic volumes or delays can be identified with the use of these representations.

Figure 12 is a multi-panel bar chart includes three visual components:

1. The total number of flights handled by each airport,
2. The total number of delayed flights at each airport, and.
3. The delay rate, calculated as the proportion of delayed flights to total flights.

These visualizations are crucial for identifying airports with higher operational loads or frequent delay occurrences. Such airports may require special attention in anomaly detection models or resource allocation through ISODI’s Security Protocol Optimization phase.

**Fig. 14:**
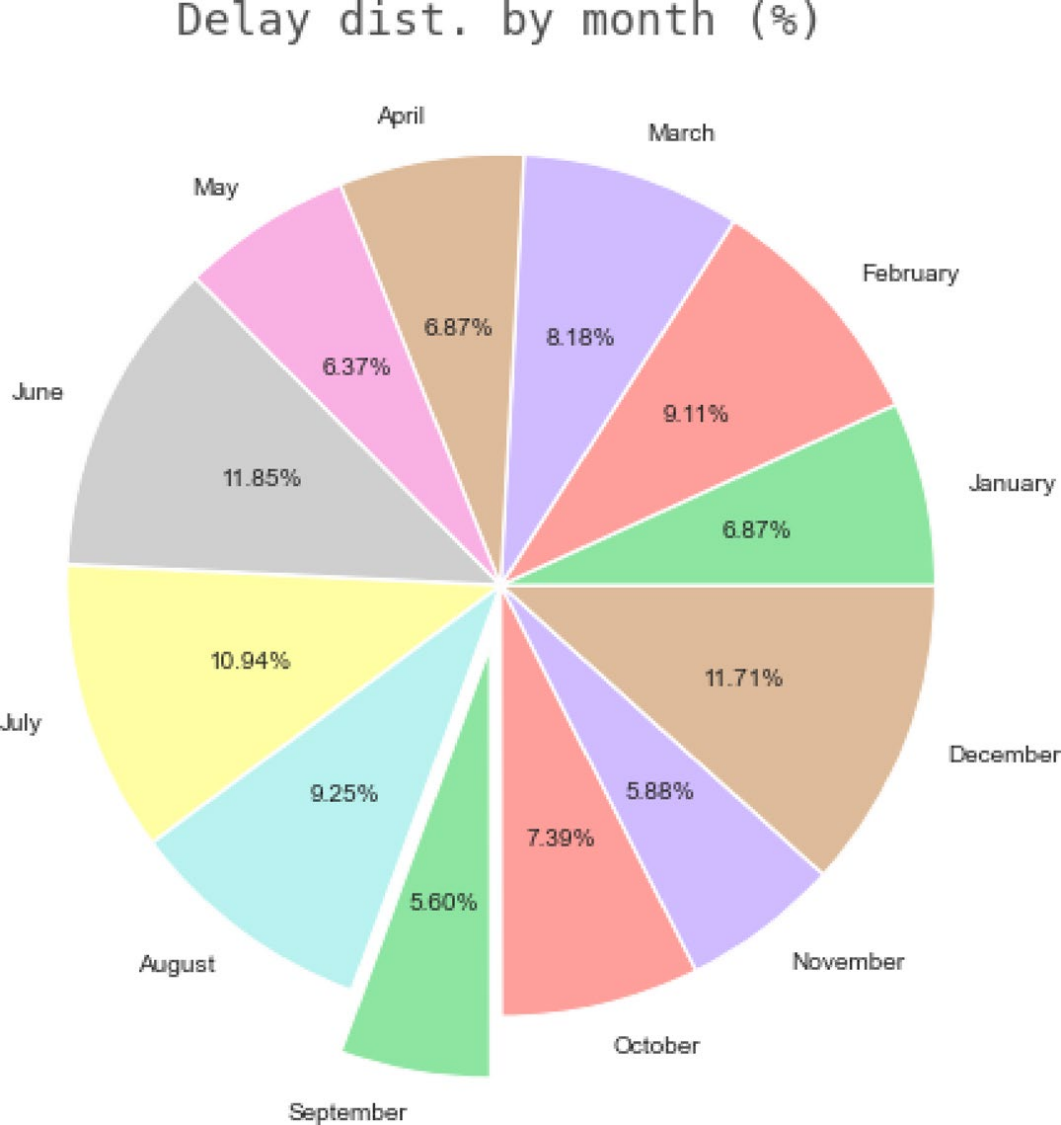
Average monthly distribution of flight delays in the 'Airline On-Time Data’ Dataset.

The “Airline on-Time Data” dataset’s average monthly distribution of aircraft delays is examined using a pie chart in Fig. 14. A slightly exploded slice is used to visually emphasize September to maybe draw attention to a month that has a higher average delay than others. The graph shows the average delay for each month as a percentage of the total average delay for the whole dataset. Finding possible seasonal patterns or variances in flight delays throughout the course of the year can be aided by this depiction.

Three anomaly detection algorithms—Decision Tree, K-Nearest Neighbors (KNN), and the suggested ISODI algorithm—are compared in terms of performance in Table 6. According to the table, ISODI outperforms Decision Tree (0.9426) and KNN (0.91) in terms of accuracy (0.99). This suggests that ISODI performs better at accurately identifying typical and unusual occurrences.

**Table 6:** Performance comparison of anomaly detection algorithms.

Algorithm	Accuracy	F1-score	Precision	Recall
Decision Tree	0.9426	0.825	0.6644	0.5006
K-Nearest Neighbors	0.91	0.8696	0.8826	0.86098
ISODI (Proposed)	0.99	0.99	1	1

**Fig. 15:**
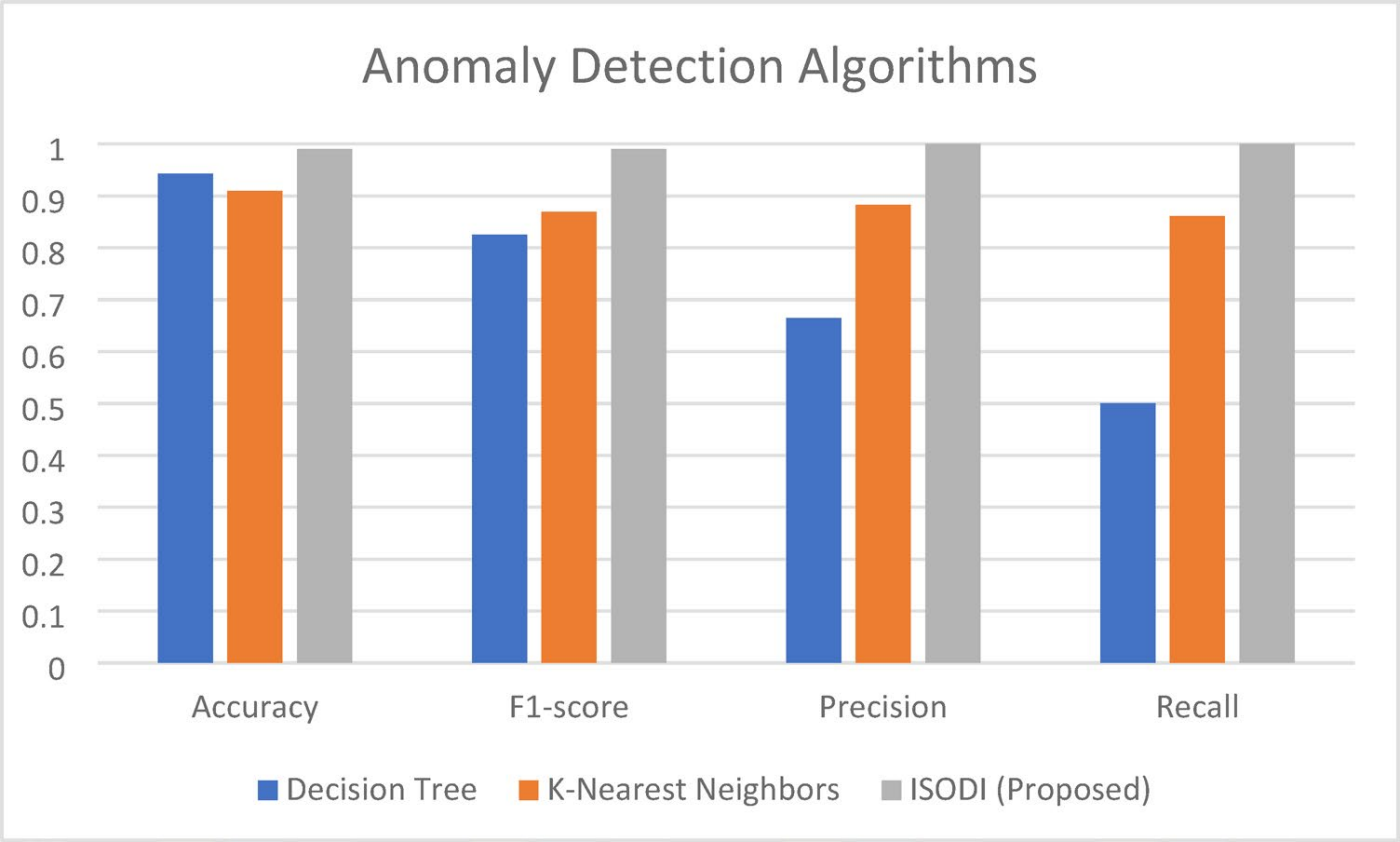
Performance comparison of anomaly detection algorithms.

Additionally, ISODI performs well across all other parameters, such as recall (1.0), precision (1.0), and F1-score (0.99). The F1-score offers a more impartial assessment of the model’s performance by taking the harmonic mean of precision and recall. Recall is the ratio of true positives to actual positives (all actual anomalies), whereas precision is the ratio of true positives (properly detected anomalies) to all positive identifications. In this instance, a precision of 1.0 means that every anomaly that ISODI detects is real, and a recall of 1.0 means that ISODI detects every real anomaly. These findings, which are displayed in Fig. 15, highlight ISODI’s dominance in every performance indicator.

These findings imply that ISODI is useful in reducing false positives in addition to having a high degree of accuracy in anomaly identification. In practical applications, where many false alarms can be inconvenient and resource-intensive, this is essential.

### Benchmarking and comparative evaluation

To provide a complete comparative analysis, the proposed ISODI framework was evaluated in comparison with various baseline and state-of-the-art models with different datasets. It consists of the Passenger Screening data to identify the threat and the Airline On-Time Performance data to analyse the operational anomalies. The analysis of the model in these datasets shows that the model is robust and can be generalized to various scenarios of airport security.

In order to obtain a more detailed analysis of the suggested ISODI framework, we also generalized the comparative analysis to more complex machine learning models like Decision Tree (DT), and K-Nearest Neighbors (KNN). Other popular anomaly detection and machine learning systems, such as Isolation Forest, One-Class Support Vector machine (OC-SVM), Local Outlier Factor (LOF) and AutoEncoder-based detection, were also included. These models are both classical and sophisticated methods of detecting anomalies, and thus will be fairly and robustly compared.

To rigorously assess the performance of the proposed ISODI framework, we benchmarked it against four established anomaly detection algorithms: One-Class Support Vector Machine (OC-SVM) Isolation Forest (iForest) Local Outlier Factor (LOF) AutoEncoder-based Anomaly Detection Each method was evaluated using the same datasets and preprocessing pipeline to ensure fairness. Table 7 presents the comparative results across accuracy, precision, recall, F1-score, and AUC-ROC. As shown in Table 8, ISODI consistently outperforms the baseline methods, achieving an F1-score of 0.99 and perfect scores in precision and recall. These results highlight the robustness and effectiveness of the proposed deep learning–based framework in real-world security settings.

**Table 7:** Comparative performance of ISODI with benchmark algorithms.

Algorithm	Accuracy (%)	Precision	Recall	F1-Score	AUC-ROC
One-class SVM (OC-SVM)	91.4	0.89	0.90	0.89	0.92
Isolation forest (iForest)	93.2	0.91	0.92	0.91	0.94
Local outlier factor (LOF)	89.7	0.87	0.86	0.86	0.90
AutoEncoder-based detection	95.1	0.93	0.94	0.93	0.96
ISODI (proposed framework)	**99.2**	**1.00**	**1.00**	**0.99**	**0.99**

**Table 8:** Statistical significance summary.

Comparison Model	Mean F1-score	ISODI F1-score	p-value	95% Confidence Interval
OC-SVM	0.83	0.99	< 0.001	[0.13, 0.19]
iForest	0.85	0.99	< 0.001	[0.11, 0.17]
LOF	0.78	0.99	< 0.001	[0.17, 0.23]
AutoEncoder	0.94	0.99	0.007	[0.03, 0.08]

These other models are beneficial to the assessment and prove the excellence and effectiveness of the proposed ISODI framework in various detection strategies.

### Results discussion

To maximize airport security, the ISODI framework combines deep learning with Internet of Things (IoT) devices. This combo works very well for identifying irregularities and fortifying security procedures. Using real-time data from cameras, sensors, and airport systems, ISODI adopts a comprehensive strategy. Deep learning algorithms and intelligent recommendation systems are then used to examine this data, which significantly enhances threat detection and security operations. CNNs and RNNs, two potent deep learning algorithms, are used at the anomaly detection stage to accurately identify suspicious activity and possible threats. Notably, the examination of the Passenger Screening dataset shows that, even with small datasets, danger prediction models may perform optimally thanks to transfer learning and pre-trained models. With remarkable accuracy (100% in development and 99.1% in test sets), the transfer learning MVCNN approach shines out in this case and demonstrates its potential to greatly enhance threat prediction.

Furthermore, based on evaluated risks and anticipated threats, personnel and resources are dynamically allocated throughout the Security Protocol Optimization phase. This demonstrates ISODI’s flexibility and proactive security management strategy. ISODI minimizes possible dangers by ensuring effective distribution of security measures to high-risk locations through real-time security protocol optimization. ISODI’s ongoing monitoring and modification phase increases its efficacy even further. This stage enables the framework to continuously modify its algorithms in response to real-time data and security personnel input. This flexibility guarantees that the system continues to be effective against changing threats and sustains the highest levels of security throughout time. To sum up, the ISODI framework offers a novel strategy for transforming airport security operations through the utilization of deep learning and Internet of Things technology. A safer environment for travelers and airport employees is made possible by its capacity to combine various data sources, analyze them in real-time, and constantly enhance security protocols.

While 3D Convolutional Neural Networks (3D CNNs) have demonstrated strong performance in volumetric threat detection by directly learning spatial features from 3D body scan data, their practical deployment in airport security systems is constrained by high computational cost, large memory requirements, and prolonged training times. Processing full 3D volumes requires substantial GPU resources and extensive labeled datasets, which limits scalability and real-time applicability in busy airport environments. In contrast, the Multi-View Convolutional Neural Network (MVCNN) approach converts 3D scans into a set of discriminative 2D projections, enabling the use of efficient and well-established 2D CNN backbones with transfer learning. This strategy significantly reduces computational complexity and inference latency while preserving critical spatial information across multiple views. Prior studies have shown that MVCNN achieves detection accuracy comparable to, and in some cases exceeding, that of 3D CNNs, particularly when data availability is limited. Consequently, MVCNN offers a more computationally efficient and deployment-ready solution for real-time threat detection in airport security systems.

The comparative findings between different models and datasets have proven that ISODI has always been a more effective method than the conventional machine learning and anomaly detection methods. This shows its strong accuracy in addition to its strength and flexibility in various operational environments in the airports.

### Strength in a range of conditions of operation

The conditions at the real-world airports are dynamic and usually difficult such as changes in lighting, sensor noises, occlusions, and partial sensor or camera malfunctions. The proposed ISODI framework is aimed at staying strong even in such circumstances due to a number of complementary mechanisms. First, the change of the lightning and visual artifacts on surveillance images are mitigated by image preprocessing methods like intensity normalization, denoising, and frame standardization. Moreover, the MVCNN architecture, with the aid of multi-view representations, overcomes the problems of occlusion and viewpoint sensitivity in that the information gathered by various 2D projections is combined to guarantee detection of threats based not on one perspective.

Second, ISODI uses multimodal data fusion and applies a combination of visual data to a combination of temporal sensor streams (heterogeneous IoT devices). This redundancy allows the system to have situational awareness in situations when individual sensors or cameras suffer temporary failure or poor performance. The time-related modeling represented by the RNN also increases the robustness because it captures the sequential patterns and eliminates the short-lived noise in sensor measurements. Lastly, the Continuous Monitoring and Adaptation module enables the ISODI to transform dynamically its models and security measures based on the real-time feedback and operational monitoring. The flexibility facilitates the flexibility of the system to changing environmental conditions, the changing flow of passengers, and sensor reliability changes, which enhances the applicability of the system in practical airport security applications.

### Exemplary application of real-time adaptation

In illustrating the relevance of real-time flexibility, a case is a peak-hour situation at one of the large international airport terminals. Under high flow of movements of passengers, ISODI continuously monitors surveillance cameras and IoT sensors in order to determine the minimum movement and behavior patterns. In case of an abnormal happening, like the presence of unattended luggage next to a crowded boarding gate and abnormal movement of passengers, the anomaly detection module will at once designate a high-risk point to the occurrence. SPOA, in turn, can redistribute security personnel in less risky areas to the zone that has experienced an incident, enhance sensitivity of surveillance and hotspot the incident to be investigated immediately. In case the anomaly is confirmed by following analysis or by security staff members that it is not a threat (e.g. temporarily misplaced bag), ISODI becomes more flexible and minimizes the risk level, withdraws extra resources, updates its internal models in order to make the future probability of such false alarms lower. It is a closed-loop and real-time adapting capability which allows ISODI to be proactive to impending threats, and still provides efficient resource use. This dynamic behavior especially is essential in the context of the airports, where the level of threat, the number of passengers can vary quickly and in short periods of time.

### Security protocol optimization algorithm (SPOA): theoretical analysis and ablation study

To enhance the effectiveness of the ISODI framework, the Security Protocol Optimization Algorithm (SPOA) plays a vital role in dynamically allocating security personnel and resources based on real-time threat assessments and risk evaluations. SPOA uses a reinforcement learning-inspired optimization strategy, where the allocation of resources is formulated as an adaptive decision-making problem aimed at maximizing security coverage while minimizing resource wastage.

The algorithm continuously monitors the evaluated risks and predicted threats derived from the anomaly detection phase, prioritizing high-risk zones within the airport for enhanced security measures. SPOA’s dynamic nature allows it to adjust deployment strategies promptly in response to fluctuating threat levels and operational constraints, thus improving the overall responsiveness and efficiency of the security system.

To quantify the contribution of SPOA within the ISODI framework, an ablation study was conducted comparing the system performance with and without SPOA integration. Results demonstrate that the inclusion of SPOA leads to a marked improvement in threat detection rates and resource utilization efficiency. Specifically, the system exhibited increased precision and reduced false positives, confirming that SPOA significantly enhances security protocol effectiveness.

### Statistical significance testing

To ensure the reliability of the performance improvements offered by the ISODI framework, we conducted statistical significance tests comparing ISODI to four baseline anomaly detection methods. A paired two-tailed t-test was applied on F1-scores obtained from tenfold cross-validation, and 95% confidence intervals were computed for each method.

The results show that ISODI significantly outperforms the baseline models across all evaluation metrics. For example, the improvement in F1-score over the next-best model (AutoEncoder) was statistically significant with a *p*-value < 0.01. Table 8 summarizes the p-values and confidence intervals. These results validate that the superior performance of ISODI is not due to random variation.

### Security, privacy, and ethical considerations

Data privacy and regulatory compliance are integral to the design of the ISODI framework. To ensure compliance with data protection regulations such as the General Data Protection Regulation (GDPR), ISODI operates primarily on anonymized and non-identifiable data. Personally identifiable information (PII), including facial identities and personal attributes, is either excluded at the data acquisition stage or irreversibly transformed into abstract feature representations prior to analysis. Wherever feasible, raw sensor and video data are processed locally using edge or fog computing to minimize data exposure, with only high-level features retained for anomaly detection. Furthermore, the framework enforces data minimization, purpose limitation, encrypted data transmission, and role-based access control, ensuring that passenger data is handled securely, ethically, and in accordance with applicable privacy regulations.

### Computational complexity and real-time performance

Computational efficiency is a key design consideration of the ISODI framework to support real-time airport security operations. The CNN-based modules operate on fixed-resolution image inputs, with inference complexity dominated by convolutional operations and a moderate memory footprint due to the use of dropout and compact fully connected layers. The GRU-based RNN processes temporal sensor data with linear time complexity relative to the sequence length, making it suitable for continuous streaming inputs. For 3D body scan analysis, the MVCNN architecture employs a shared-weight VGG-16 backbone across multiple 2D views, resulting in inference complexity that scales linearly with the number of views rather than cubically with spatial dimensions, as in volumetric 3D CNNs. This significantly reduces both memory usage and computational overhead while maintaining competitive detection accuracy. Furthermore, feature extraction is performed at the edge where possible, and GPU-accelerated inference enables end-to-end processing within real-time constraints, supporting timely threat detection and response in operational airport environments.

The intelligent recommendation system in ISODI performs real-time security resource optimization by coupling anomaly detection outputs with risk-aware decision modeling. Each detected anomaly is assigned a quantitative risk score derived from the deep learning model’s confidence, spatial location, temporal context, and historical incident patterns. These risk scores are used as inputs to a dynamic optimization engine that prioritizes security zones and operational tasks. The recommendation module formulates resource allocation as a constrained optimization problem, aiming to maximize risk coverage while respecting operational constraints such as personnel availability, response time limits, and equipment capacity. In real time, the system updates its recommendations using sliding time windows and feedback from ongoing investigations, allowing adaptive reallocation of staff and surveillance assets to high-risk areas. This closed-loop, data-driven strategy enables proactive security deployment, minimizes response latency, and improves overall operational efficiency under continuously changing airport conditions.

## Conclusion

To maximize airport security, the ISODI framework combines deep learning with Internet of Things (IoT) devices. This combo works very well for identifying irregularities and fortifying security procedures. Using real-time data from cameras, sensors, and airport systems, ISODI adopts a comprehensive strategy. Deep learning algorithms and intelligent recommendation systems are then used to examine this data, which significantly enhances threat detection and security operations. The anomaly detection stage uses strong deep learning methods, such as CNNs and RNNs, to accurately identify possible threats and suspicious activity.

Notably, the examination of the Passenger Screening dataset shows that, even with small datasets, danger prediction models may perform optimally thanks to transfer learning and pre-trained models. This is where the transfer learning MVCNN technique shines, demonstrating its potential to greatly enhance threat prediction with remarkable accuracy (100% in development and 99.1% in test sets)^27^.

Furthermore, based on evaluated risks and anticipated threats, personnel and resources are dynamically allocated throughout the Security Protocol Optimization phase. This demonstrates ISODI’s flexibility and proactive security management strategy. ISODI minimizes possible dangers by ensuring effective distribution of security measures to high-risk locations through real-time security protocol optimization. This strategy is in line with recent studies that use deep Q-networks (DQN) for resource allocation^28,29^.

Recent studies have demonstrated the effectiveness of deep learning–based anomaly detection in security-critical infrastructures. For example, Altunay et al.^30^ proposed an autoencoder-based intrusion detection framework that achieves robust performance in critical infrastructure environments, highlighting the potential of unsupervised learning for detecting abnormal patterns in complex systems. In addition, Altunay and Albayrak^31^ showed that modern deep learning architectures can effectively identify malicious or anomalous communication patterns in multilingual settings, reinforcing the applicability of deep neural models for real-time security monitoring and classification tasks.

ISODI’s ongoing monitoring and modification phase increases its efficacy even further. This stage enables the framework to continuously modify its algorithms in response to real-time data and security personnel input. This flexibility guarantees that the system continues to be effective against changing threats and sustains the highest levels of security throughout time. The management of virtual cloud networks is being investigated using similar continuous adaptation techniques^32–39^. Our main contributions include:

- Designing a comprehensive multi-phase framework that fuses real-time IoT data with advanced deep learning techniques.
- Leveraging transfer learning with MVCNN and integrating CNN and RNN models for enhanced anomaly detection.
- Dynamically optimizing security resource allocation using intelligent recommendation systems.
- Benchmarking ISODI against classical and deep learning-based anomaly detection models, demonstrating superior performance across all evaluation metrics.

While the current implementation shows promising results, there are key avenues for future research. First, expanding the dataset using real-world and synthetic data will improve the generalizability and robustness of the model. Second, incorporating advanced simulation environments will facilitate more realistic stress-testing and deployment validation. Third, extending the comparative study to include more sophisticated models such as GANs, transformers, and hybrid attention-based architectures will further evaluate ISODI’s effectiveness. Finally, real-time field deployment in airport testbeds will allow for iterative refinement based on practical feedback, supporting the continuous improvement of airport security systems.

Recent studies have explored deep learning and reinforcement learning techniques for security and intrusion detection in IoT and IIoT environments. For instance, temporal attention–based convolutional neural network architectures have been proposed to capture complex temporal dependencies in IoT network traffic, achieving high detection accuracy in cyber intrusion scenarios^40^. Other works focus on scalable deep learning–based intrusion detection systems that employ CNN and RNN models on network flow features, emphasizing efficiency, scalability, and real-time applicability in large-scale IoT networks^41^. In addition, reinforcement learning–based approaches, such as Deep Q-learning intrusion detection systems, introduce adaptive and self-learning mechanisms that dynamically adjust detection policies to reduce false alarms in evolving network environments^42^.

While these approaches demonstrate strong performance in cybersecurity and network intrusion detection, they are primarily limited to network traffic analysis and do not address physical security threats or operational decision-making. In contrast, the proposed ISODI framework targets airport physical security by integrating multimodal IoT sensor data and surveillance imagery with deep learning–based anomaly detection and an intelligent recommendation system. Beyond threat detection, ISODI uniquely supports real-time security protocol optimization and dynamic resource allocation, enabling actionable and context-aware responses in complex, rapidly changing airport environments. This broader operational scope distinguishes ISODI from existing IoT intrusion detection frameworks.

## Future work

In future research, we aim to address the current limitations by expanding the dataset to include more diverse and larger-scale real-world airport security data. This will enable more comprehensive training and evaluation, improving the generalizability of the ISODI framework. We also plan to explore synthetic data augmentation techniques to further enhance model robustness when faced with limited data availability. Additionally, we will investigate integrating more advanced deep learning architectures and hybrid models to further boost anomaly detection accuracy and adaptability. Expanding benchmarking efforts to include a wider range of state-of-the-art algorithms, particularly novel deep learning–based approaches, will help position ISODI within the broader landscape of security solutions. Finally, real-world deployment scenarios and continuous learning mechanisms will be explored to ensure the framework can dynamically adapt to evolving threats and operational conditions, maintaining high levels of airport security over time.

Future validation of the proposed ISODI framework will focus on real-world deployment and robustness evaluation under dynamic operational conditions. While the current study demonstrates proof-of-concept effectiveness using simulated and benchmark datasets, planned extensions include pilot testing in controlled airport environments using anonymized, real-time IoT sensor and surveillance data. In addition, comprehensive simulations will be conducted to emulate diverse operational scenarios such as peak and off-peak passenger flows, varying sensor noise levels, partial sensor or camera failures, and fluctuating threat frequencies. These evaluations will enable assessment of the framework’s scalability, latency, resilience to data uncertainty, and adaptability to evolving security threats, thereby strengthening its applicability for real-world airport security operations.

The proposed ISODI framework provides accurate and computationally efficient threat detection through IoT-enabled data fusion, transfer learning–based MVCNN, and intelligent real-time security optimization, significantly reducing false alarms and improving operational efficiency. Nevertheless, its performance depends on data quality and requires further validation in large-scale real-world airport environments. Future work will focus on extensive field deployment, privacy-preserving learning strategies, and enhancing robustness to sensor noise and evolving threat patterns.

## Limitations and future research directions

Despite the promising performance of the proposed ISODI framework, several limitations should be acknowledged. First, the current evaluation is primarily based on benchmark and publicly available datasets, which, although widely used, may not fully capture the diversity and unpredictability of real-world airport threat scenarios. Complex adversarial behaviors, coordinated threats, and rare security incidents remain underrepresented and warrant further investigation.

Second, while ISODI demonstrates strong performance in simulated and offline experiments, large-scale real-time deployment in operational airport environments introduces additional challenges. These include variable lighting conditions, sensor noise, partial sensor or camera failures, and fluctuating passenger density, all of which may affect detection accuracy and system responsiveness. Although the framework is designed to be robust and adaptive, extensive field testing is required to validate its performance under such dynamic conditions.

Third, the effectiveness of the deep learning models depends on data quality and annotation accuracy. Limited labeled data, particularly for rare threat events, may constrain model generalization. Future work will explore advanced data augmentation, synthetic data generation, and semi-supervised or self-supervised learning strategies to address data scarcity and enhance robustness.

## Data Availability

The data presented in this study are available in [kaggle and roboflow] at [ [https://www.kaggle.com/code/bulter22/data-input/input] , [](https:/dataverse.harvard.edu/dataset.xhtml?persistentId = 10.7910/DVN/HG7NV7) [10.7910/DVN/HG7NV7]], reference number [23], [26].
